# Size, shape, and form: concepts of allometry in geometric morphometrics

**DOI:** 10.1007/s00427-016-0539-2

**Published:** 2016-04-01

**Authors:** Christian Peter Klingenberg

**Affiliations:** Faculty of Life Sciences, University of Manchester, Michael Smith Building, Oxford Road, Manchester, M13 9PT UK

**Keywords:** Allometry, Centroid size, Conformation, Form, Geometric morphometrics, Multivariate regression, Principal component analysis, Procrustes superimposition, Shape, Size correction

## Abstract

Allometry refers to the size-related changes of morphological traits and remains an essential concept for the study of evolution and development. This review is the first systematic comparison of allometric methods in the context of geometric morphometrics that considers the structure of morphological spaces and their implications for characterizing allometry and performing size correction. The distinction of two main schools of thought is useful for understanding the differences and relationships between alternative methods for studying allometry. The Gould–Mosimann school defines allometry as the covariation of shape with size. This concept of allometry is implemented in geometric morphometrics through the multivariate regression of shape variables on a measure of size. In the Huxley–Jolicoeur school, allometry is the covariation among morphological features that all contain size information. In this framework, allometric trajectories are characterized by the first principal component, which is a line of best fit to the data points. In geometric morphometrics, this concept is implemented in analyses using either Procrustes form space or conformation space (the latter also known as size-and-shape space). Whereas these spaces differ substantially in their global structure, there are also close connections in their localized geometry. For the model of small isotropic variation of landmark positions, they are equivalent up to scaling. The methods differ in their emphasis and thus provide investigators with flexible tools to address specific questions concerning evolution and development, but all frameworks are logically compatible with each other and therefore unlikely to yield contradictory results.

## Introduction

Variation in size is an important determinant for variation in many other organismal traits. Developmental processes are accompanied by a dramatic growth in size in developing organisms, and evolutionary diversification often involves differentiation of body size among related taxa. Accordingly, allometry has been an important concept for evolutionary biology and related disciplines for much of the last century (Huxley 1924, 1932; Cock 1966; Gould 1966; Calder 1984; Schmidt-Nielsen 1984). During this time, the methods for quantifying morphological variation underwent momentous change, from the development of multivariate approaches to the emergence of the discipline of morphometrics (Jolicoeur and Mosimann 1960; Jolicoeur 1963; Sneath and Sokal 1973; Oxnard 1974; Pimentel 1979; Reyment et al. 1984). Finally, the rise of geometric morphometrics in the 1980s and 1990s has established the current methods for analyzing variation in organismal shape (Bookstein 1986, 1991; Rohlf 1990; Rohlf and Bookstein 1990; Marcus et al. 1993, 1996; Rohlf and Marcus 1993; Monteiro and dos Reis 1999; Klingenberg 2010; Zelditch et al. 2012; Adams et al. 2013; Mitteroecker et al. 2013). Throughout this history of different frameworks for quantifying morphological variation, allometry has played a more or less prominent role.

Along with the ways of characterizing morphological variation in general, the concept of allometry and the methods for analyzing it have changed drastically as well. In some approaches, the main emphasis is on covariation among different traits (Huxley 1924, 1932; Jolicoeur 1963), whereas others focus on the covariation between size and shape (Mosimann 1970; Monteiro 1999). Another difference is whether the methods separate size and shape (Mosimann 1970; Bookstein 1986; Goodall 1991) or whether they reject this distinction and consider morphological form as a single unified feature (Lele and Richtsmeier 1991; Mitteroecker et al. 2004). As a consequence of the different concepts of allometry, the various methods also differ in the way in which they carry out corrections for the effects of size on morphological variation, which is one of the most used applications of allometry (Burnaby 1966; Sidlauskas et al. 2011).

A number of review papers have provided overviews of the biological concepts related to allometry (Cock 1966; Gould 1966; Klingenberg 1998) and the statistical methods for allometric analyses mainly in the context of traditional morphometrics (Bookstein 1989; Klingenberg 1996b). There is no comparable survey, however, for allometric analyses in the context of geometric morphometrics (but see Mitteroecker et al. 2013). This paper surveys the methods for analyzing allometry in geometric morphometrics. To appreciate the range of current concepts and their interrelations, it is helpful to take a historical perspective that considers the origin of ideas before explaining their role in currently used methods in geometric morphometrics. This article adopts this approach and therefore starts by revisiting the concepts of allometry, size, and shape as they have been used traditionally, before employing these concepts to compare the different frameworks that are currently used in geometric morphometrics. Considering the structure of shape spaces has proven helpful for comparing morphometric methods (Rohlf 1996, 2000) and serves in this paper as a framework for comparing different methods for analyzing allometry. A particular focus of attention is how the different allometric concepts are applied for size correction in morphometric data. Finally, the relationships among alternative methods are discussed.

## Allometry, size, and shape in different morphometric frameworks

There are several concepts of allometry, which all concern the effect of size on morphological variation, but differ in the specific definitions of terms and in the aspects of morphology on which they focus. It is possible to distinguish two main schools of thought according to the way they characterize allometry: the Huxley–Jolicoeur school, which emphasizes the covariation among traits as a consequence of variation in size, and the Gould–Mosimann school, which defines allometry as covariation of size and shape (Klingenberg 1998). A fundamental difference between the two concepts of allometry is that the framework of the Huxley–Jolicoeur school does not involve a distinction of size and shape, which is the central element in the framework of the Gould–Mosimann school.

As it turns out, the distinction between these two schools is also useful for understanding the differences between different allometric approaches currently used in geometric morphometrics. Therefore, this section provides a brief overview of the two schools of thought and contrasts them directly with each other in some key aspects. The purpose of this section is solely to provide a background for the comparison of the methods currently used in geometric morphometrics. It is therefore not a complete historical survey of allometry, but inevitably leaves out many concepts and methods.

A common feature of both schools of thought is the treatment of size. In all the different frameworks, allometry is variation in various traits that is explained by or associated with variation in size (Gould 1966; Mosimann 1970; Bookstein et al. 1985; Klingenberg 1998; Mitteroecker et al. 2013). The origin of the size variation depends on the context of the study, and according to this context, different levels of variation can be defined (Cock 1966; Gould 1966; Cheverud 1982; Klingenberg and Zimmermann 1992a; Klingenberg 2014). Many studies have analyzed the changes associated with the dramatic size increases over individual growth, or ontogenetic allometry (Huxley 1924, 1932; Loy et al. 1996; Bulygina et al. 2006; Rodríguez-Mendoza et al. 2011; Mitteroecker et al. 2013; Murta-Fonseca and Fernandes 2016). Others have focused on the consequences of size variation within a single ontogenetic stage, or static allometry, most often based on samples of adults from a population (Rosas and Bastir 2002; Drake and Klingenberg 2008; Weisensee and Jantz 2011; Freidline et al. 2015). Evolution can also alter the size of organisms and produce associated morphological changes due to evolutionary allometry (Cardini and Polly 2013; Klingenberg and Marugán-Lobón 2013; Martín-Serra et al. 2014; Sherratt et al. 2014). These three levels of allometry, and sometimes others as well, have been compared in a range of studies (Cheverud 1982; Leamy and Bradley 1982; Klingenberg and Zimmermann 1992a; Klingenberg et al. 2012; Pélabon et al. 2013; Freidline et al. 2015; Strelin et al. 2016). These three classical levels of allometry are not the only levels of variation where allometry can apply, but others exist as well and may be worth investigating (Klingenberg 2014). For example, fluctuating asymmetry of shape may have a component of allometry, where asymmetry of shape is an allometric consequence of asymmetry in size (Klingenberg 2015). Such allometry of fluctuating asymmetry has been found in several study systems (e.g., Klingenberg et al. 2001; Breuker et al. 2006; Ludoški et al. 2014; Martínez-Vargas et al. 2014). The different allometric frameworks reviewed in this paper are applicable to all these levels—the level of allometry reflects the composition of the data, whether it is a single growth stage or an ontogenetic series, a single population or multiple species from some clade.

If a dataset contains more than one source of size variation, and thus more than one level of allometry, problems can arise because the levels of allometry may be confounded. For instance, if there is ontogenetic variation as well as environmental or genetic variation that affects size, there is ontogenetic allometry and possibly also allometry in response to the genetic and environmental variation. To disentangle these levels, study designs are required in which the different factors are known, and the analyses of allometry need to reflect those designs. For instance, if genotypes and environments are known, they can be used as grouping criteria and pooled within-group analyses for the different levels of allometry can be carried out. If the factors causing size variation are not known explicitly, such a separation of levels is not possible and they will be inevitably confounded to a greater or lesser extent.

### Bivariate and multivariate allometry: the Huxley–Jolicoeur school

**Fig. 1:**
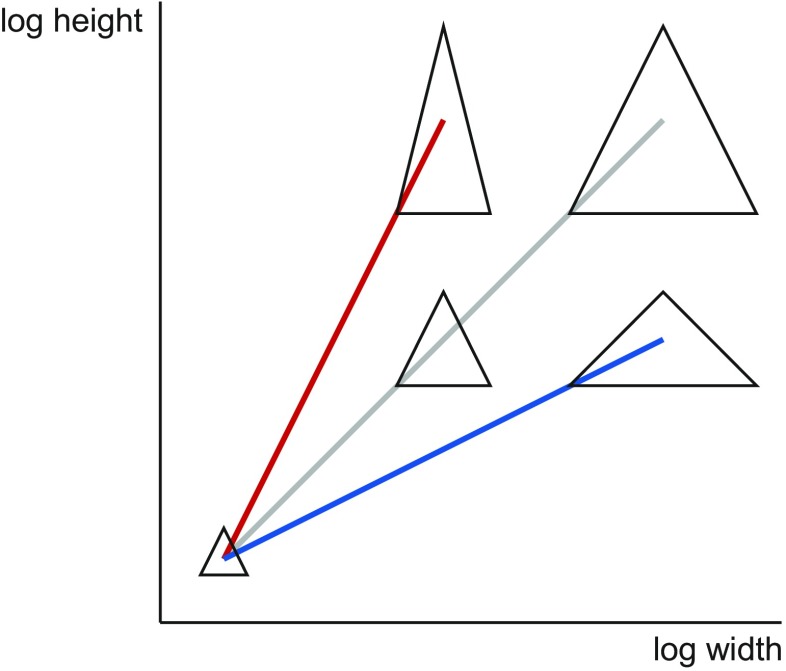
Bivariate allometry of height versus width in a set of triangles. The *gray line* shows isometry, with a slope of 1.0 in the plot of log-transformed height versus width and no change in the ratio of the two measurements. The *red line* is an example of positive allometry of height versus width, and the *blue line* is an example of negative allometry

The concept of allometry originated from the discovery that pairwise plots of log-transformed length measurements of organisms often fit remarkably well to straight lines and the idea that constant ratios between the relative growth rates of different parts can account for this (Huxley 1924, 1932). The slopes in pairwise plots of log-transformed measurements (Fig. 1) indicate the ratios between the relative growth rates of the respective traits and are the basis for the terminology of allometry (Huxley and Teissier 1936). Usually, one trait is considered as the trait of interest and is plotted on the vertical axis, whereas the other trait is considered as a measure of size and is plotted on the horizontal axis. If both traits have the same relative growth rates, the slope in plots of log-transformed traits is 1.0 and their proportions do not change as size increases, a situation that is called isometry (gray line in Fig. 1). If the trait of interest has the greater relative growth rate, it is increasing disproportionately more in relation to the size measure, so that the slope is greater than 1.0—a situation that is called positive allometry (red line in Fig. 1). Conversely, for negative allometry, the trait of interest has a lesser relative growth rate than the size measure, it is increasing disproportionately less than the size measure and the slope is less than 1.0 (blue line in Fig. 1).

**Fig. 2:**
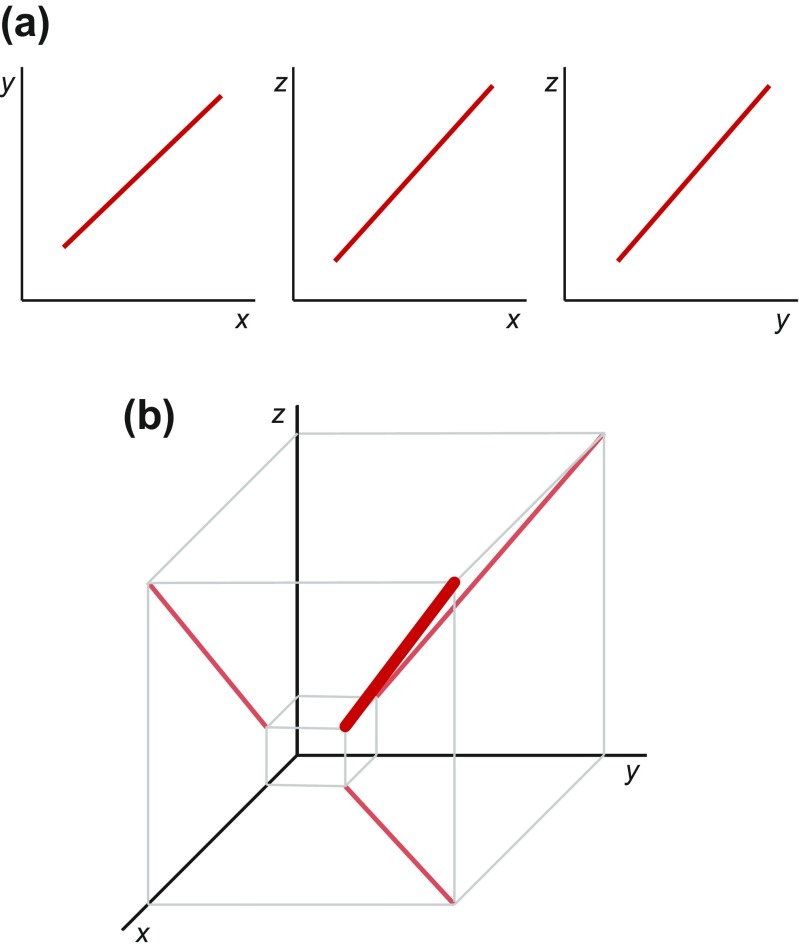
Bivariate and multivariate allometry. **a** Three pairwise plots of three variables (e.g., log-transformed measurements). In each plot, there is a linear allometric relationship (*red line*). **b** Combining the three bivariate relationships into a 3D space. The allometric trajectory is a line in the space (*bold red line*), whereas the bivariate plots are its projections onto the planes defined by pairs of axes (*pale red lines*). Note that all three variables are equivalent and there is no basis for choosing “dependent” or “independent” variables (modified from Klingenberg 1998)

If there are more than two traits, a multivariate generalization of this allometry concept is required because considering all pairwise plots of variables may become very cumbersome even for relatively few measurements. The most straightforward such generalization considers a multidimensional space where each log-transformed measurement corresponds to one axis. Each bivariate allometric plot is then a projection from this multidimensional space onto the plane defined by the axes that correspond to the two measurements (Fig. 2a). From this line of reasoning, it follows that the multivariate generalization of the straight lines in bivariate allometric plots is a straight line in the space of log-transformed measurements (Fig. 2b). The question then arises how to estimate this line in the multivariate space.

Jolicoeur (1963) offered a solution for this problem: run a principal component analysis (PCA) of the covariance matrix of log-transformed measurements and use the resulting first principal component as the estimate of the allometric line. Because the first principal component (PC1) is that direction in the multidimensional space that accounts for the greatest possible proportion of the total variance, the sum of the variances in all the directions perpendicular to it is minimal. This use of principal components follows the original proposal of the method (not yet under that name) as a means to obtain a best-fitting line for multivariate data according to a least-squares criterion (Pearson 1901). PCA is one of the most fundamental and widespread methods in multivariate statistics (Jolliffe 2002) and is widely used for studying multivariate allometry (for review, see Klingenberg 1996b). Using the PC1 to estimate a best-fitting line is very suitable for multivariate allometry, because it treats all variables equally.

The coefficients of the PC1 indicate its direction in the multidimensional space (the PC1 coefficient of each variable can be interpreted as the cosine between the coordinate axis for that variable and the PC1 axis). The bivariate allometric coefficients for specific pairs of variables can be obtained from the ratios of the respective PC1 coefficients (Jolicoeur 1963). If the data fit a straight line tightly, the bivariate and multivariate estimates are usually close, but if there is a substantial amount of scatter, they may differ (depending also on the specific method used to obtain the bivariate coefficients). For isometry, with no change in the proportions among variables along the allometric axis, all PC1 coefficients are equal—with the usual scaling for PC coefficients, for *p* variables each PC1 coefficient is then *p*^−0.5^ (Jolicoeur 1963). Some empirical studies have examined the results of bivariate and multivariate allometry and generally found a close agreement (e.g., Davies and Brown 1972; Shea 1985).

A key assumption of the Huxley–Mosimann approach is that size is the dominant contributor of variation in the measurements and of covariation between them. If this is not the case, for instance in data from organisms that are strictly standardized for size, bivariate regressions or the PC1 will not reflect allometry, but some other factor. To guard against this possibility, investigators should check that there is indeed a noticeable amount of size variation in the data, which is obvious in many instances. Furthermore, the coefficients from regressions or PCA can indicate whether the data are consistent with the expectations for allometry. Because allometric variation usually means that all measurements increase together, although possibly some more than others, it is expected that all bivariate regression coefficients are positive and all PC1 coefficients have the same sign (also usually positive). This test is not absolutely reliable, because there are rare examples where one or a few measurements decrease with overall size increase (a phenomenon called enantiometry; Huxley and Teissier 1936; Klingenberg 1996b). In the vast majority of biological datasets, however, size is the dominant factor contributing to variation and allometric analyses using the Huxley–Jolicoeur approach are therefore appropriate.

The position along the allometric axis can be used as an indication of the overall size of each specimen (but note that the allometric axis is usually associated with changes in shape, so this index will usually be correlated with shape in various ways). In practice, this means that the PC1 scores, or PC1 scores rescaled so that they behave as a linear dimension (Klingenberg and Zimmermann 1992b), can be used as an index of size that takes into account all measured traits simultaneously (Creighton and Strauss 1986; Klingenberg and Spence 1993; Klingenberg 1996a).

**Fig. 3:**
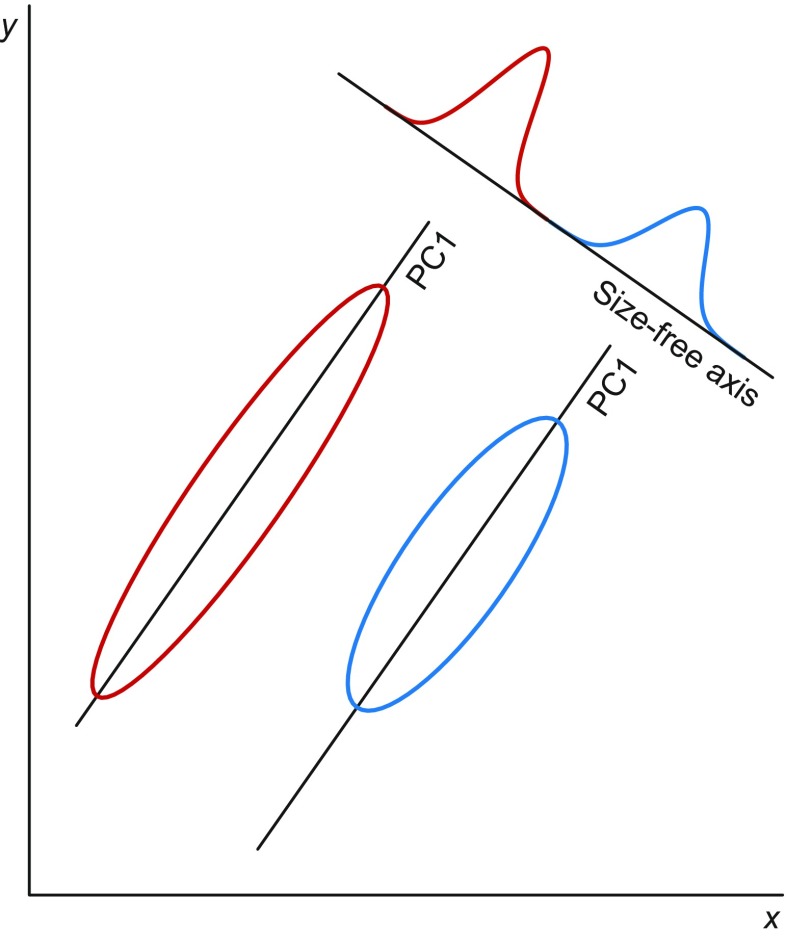
Size correction in the Huxley–Jolicoeur framework. The first principal component (PC1) in each group is an allometric axis. Projecting the data onto the space perpendicular to this allometric axis yields a space free of size and size-associated morphological variation (Burnaby 1966). In this bivariate example, this is a single size-free axis. For multiple groups, the assumption is that the allometric axes of all groups are parallel

By contrast, the directions perpendicular to the allometric axis correspond to variation that is uncorrelated to size and morphological changes related to size via allometry, and therefore constitute a size-free axis or space (Fig. 3). Using this size-free space for further analyses is a way to correct for allometric effects of size. Burnaby (1966) provided a method to project the data onto a space orthogonal to one or more arbitrary vectors, usually an allometric vector, which has been widely adopted and discussed (Rohlf and Bookstein 1987; Klingenberg 1996b; McCoy et al. 2006). If the PC1 of log-transformed measurements is used as an allometric vector, as is often done, Burnaby’s procedure reduces to omitting the PC1 and only using the other PCs in further analyses. This is often very effective: in the example of Fig. 3, where the groups overlap for each of the variables considered separately, the size-free axis can clearly distinguish the two groups.

If there are two or more groups in the data, Burnaby’s method for size correction requires that the allometric axes of the groups are parallel (Fig. 3) or, at least, that the directions of the allometric axes are sufficiently similar so that a single direction can be found that is a satisfactory compromise for all groups. There are generalizations of PCA for estimating the common allometric axis in multigroup studies: multigroup PCA (MGPCA; Pimentel 1979; Thorpe 1983), which is a PCA of the pooled within-group covariance matrix, and the somewhat more flexible method of common principal component analysis (CPCA; Airoldi and Flury 1988; Flury 1988), which is based on a model where all groups share the same directions of the PCs, but may differ in the amounts of variation for which each PC accounts. In the context of allometry, numerous studies have used either MGPCA (Leamy and Thorpe 1984; Smith and Patton 1988; Lessa and Patton 1989; Jones 1992; Malhotra and Thorpe 1997) or CPCA (Klingenberg and Zimmermann 1992a; Klingenberg and Spence 1993; Klingenberg 1996b; Klingenberg and Ekau 1996; Patterson et al. 2001; Larson 2004; Fadda and Leirs 2009; Bolzan et al. 2015).

For comparing allometries among different taxa, there are a number of graphical displays for allometric vectors that can identify growth gradients and similar patterns (Solignac et al. 1990) or the arrangement of allometric trajectories through the space of morphological variables (Boitard et al. 1982). To quantify the difference between allometric vectors, the angles between them in the multidimensional space can be computed (Boitard et al. 1982; Cheverud 1982; Gibson et al. 1984; Klingenberg and Zimmermann 1992a; Klingenberg 1996b; Klingenberg and Ekau 1996). Also, a useful strategy is to carry out an ordination analysis of allometric vectors, sometimes called an “allometric space,” for instance by using the vectors of PC1 coefficients as the observations in a PCA or other multivariate analysis (Solignac et al. 1990; Klingenberg and Froese 1991; Klingenberg and Spence 1993; Gerber et al. 2008; Wilson and Sánchez-Villagra 2010; Wilson 2013).

It is helpful to note that the framework of multivariate allometry distinguishes between components of variation according to their directions: along allometric axes and perpendicular to them. These allometric and size-free components of variation have sometimes been interpreted in terms of size and shape. Recall, however, that the framework of multivariate allometry does not include an explicit concept of shape (Klingenberg 1996b), although the term “shape” sometimes has been used informally in interpretations of the results from such analyses. Multivariate allometry, as part of the Huxley–Jolicoeur school, focuses of the covariation among measured traits but does not explicitly refer to size or shape. The direction along the allometric axis is a measure of size but also involves the part of shape change that is due to allometry. Variation in directions perpendicular to the allometric axis is therefore not the complete variation of shape and does not have a straightforward interpretation in terms of the geometry of the morphological structure under study. For the sake of clarity, therefore, it seems best to avoid the term “shape” altogether in the context of multivariate allometry (Klingenberg 1996b).

### Geometric reasoning in traditional morphometrics: the Gould–Mosimann school

A very different approach was proposed by Mosimann (1970), who offered explicit geometric definitions of size and shape and developed an analytical framework from those definitions. Size indicates the overall dimension or scale of an object. Size is a scalar property that can be quantified by a single number (but there may be different ways to calculate the size of a given object, resulting in different values). Shape is conceptually distinct from size: the shapes of two objects are equal if they are geometrically similar, regardless of the sizes of the objects. For data consisting of length measurements, this means that all measurements in two objects with identical shapes differ only by a constant factor that relates to the relative sizes of the objects. The analysis of ratios of measurements versus overall size is therefore useful for quantifying shape. In other words, shape is about the proportions of objects.

In this situation, shape can be quantified as a vector of ratios: each of the measurements divided by a standard size variable that quantifies the overall size of the object. A standard size variable, as defined by Mosimann (1970), is a positive real-valued function *G*(**x**) of the vector **x** of measurements so that the equation *G*(*c***x**) = *cG*(**x**) holds for any positive constant *c*, which means that multiplying each measurement by the factor *c* results in a *c*-fold increase in the value of the size variable. This implies that a standard size variable is a function that scales linearly in relation to the original measurements. Examples of standard size variables are any one of the original measurements, the geometric or arithmetic mean of the measurements or any linear combination of log-transformed measurements for which the coefficients sum up to 1.0.

The vector of ratios that describes shape can then be written as **x**/*G*(**x**). In practice, size and shape variables are often computed from log-transformed measurements, producing log-size and log-shape variables (Mosimann and James 1979; Darroch and Mosimann 1985). These variables, on a logarithmic scale, are comparable to those used in bivariate and multivariate allometry in the Huxley–Jolicoeur framework.

**Fig. 4:**
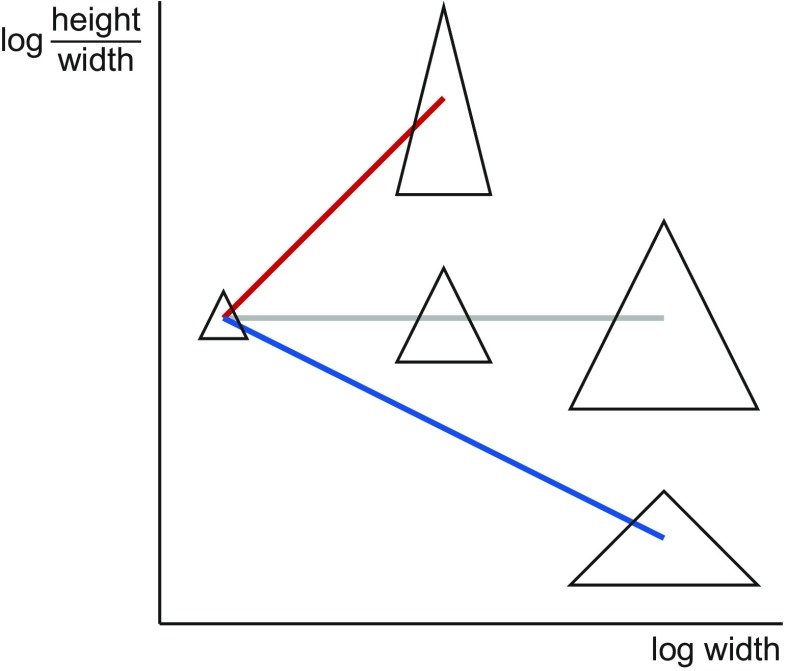
Allometry in the framework of Mosimann (1970). The horizontal axis shows the size variable (the width of the triangle) and the vertical axis a size variable (the ratio height/width). For easier comparison with Fig. 1, both variables are shown on logarithmic scales (as log-size and log-shape variables). Allometry is an association of shape with size (*red* and *blue lines*). Because the shape variable and the size measure in this example are directly related to the variables in Fig. 1, positive and negative allometry in the Huxley framework (Fig. 1) translate to an increase or decrease of the shape variable with increasing size. Isometry is the condition when there is no shape change associated with size, and the *gray line* is therefore flat

Allometry is characterized as the association between a standard size variable and the corresponding vectors of shape ratios or between a log-size variable and log-shape variables (Fig. 4). Isometry, by contrast, is the condition where size and shape are independent of each other and usually serves as the null hypothesis in tests for allometry. The question whether there is allometry or isometry can be addressed statistically by tests of multiple correlation (Mosimann 1970; Mosimann and James 1979).

Discussions about size correction need to take into account the goals of the studies that proposed the various methods. In the context of the Gould–Mosimann school, the main interest usually has been to separate size and shape per se, rather than to remove the allometric consequences of variation in size. Accordingly, a range of methods are available that provide shape data after different algebraic maneuvers that provide equivalent results, such as doubly centering the data matrix of log-transformed length measurements both by rows and columns or by projecting the data onto the subspace perpendicular to the isometric vector (Burnaby 1966; Mosimann and James 1979; Darroch and Mosimann 1985; Kazmierczak 1985; Berge and Kazmierczak 1986; Somers 1986; Rohlf and Bookstein 1987; Jungers et al. 1995; Mardia et al. 1996; Baur and Leuenberger 2011). These methods produce variables that represent the shapes of the specimens, but do not remove the allometric effects of size variation, which is why the new shape variables tend to be correlated with size. Such correlations have been the target for criticism but are inevitable if a definition of shape based on geometric similarity is used and there is allometry in the data (Cadima and Jolliffe 1996; Mardia et al. 1996).

### Comparison: size as a factor intrinsic or extrinsic to a morphospace

The preceding summaries of the allometry concepts used in the Huxley–Jolicoeur and Gould–Mosimann frameworks show some fundamental differences but also some connections. Both differences and relationships between the two frameworks can be helpful for understanding the methods currently used for studying allometry in geometric morphometrics.

The most fundamental difference is in how size relates to the morphological descriptors that are the focus of the two frameworks. Size and shape (in a geometric sense) are not separated in the Huxley–Jolicoeur framework because size is intrinsic in every morphological variable, usually length measurements, whereas the geometric distinction between size and shape is at the core of all analyses in the Gould–Mosimann school. This difference between approaches has an important consequence for the analysis of allometry. In the Huxley–Jolicoeur framework, allometry is the covariation among morphological variables due to the joint effects that size has on all of them and allometric patterns can be characterized by a line of best fit in the space of log-transformed measurements. In the Gould–Mosimann framework, by contrast, allometry manifests itself as a correlation between size and shape. The correlation approach can only be used because size and shape are logically distinct, so that it is a sensible question to ask whether there is a statistical association between them.

Besides these differences, however, there are also various connections between Mosimann’s (1970) approach and bivariate or multivariate allometry (and therefore between the Gould–Mosimann and Huxley–Jolicoeur schools). These are clearest when log-shape and log-size variables are used and they depend on the choice of the size variable. One connection can be seen by comparing Figs. 1, 2, 3, and 4: because both use the same measurement as the horizontal axis (as the size variable in the Mosimann approach, Fig. 4), they differ just by a shearing of the whole diagram, and the slope of each of the lines in Fig. 4 is the slope of the corresponding line in Fig. 1 minus 1.0 (this holds precisely if there is a perfect fit of the lines, but becomes more complicated if there is scatter around them). Further, Mosimann (1970) showed that the conditions for isometry in the two frameworks are equivalent: if the PC1 of the covariance matrix of log-transformed measurements has coefficients that are the same for all the variables, then the standard size variable computed as the geometric mean of the measurements is independent of the corresponding shape vector. Therefore, even though the Gould–Mosimann and Huxley–Jolicoeur schools differ in important ways, both frameworks agree in that allometry implies that variation in size is accompanied predictably by variation in proportions among measurements, whereas isometry means that size can change without predictable effects on proportions.

## Geometric morphometrics

The advent of geometric morphometrics in the 1980s and early 1990s brought about a series of changes of fundamental importance for the study of allometry. Ironically, one of the changes was that little attention was paid to allometry, except for some tests against the null hypothesis of isometry (Bookstein 1991). To some extent, this is understandable in the tradition of the Gould–Mosimann school and its emphasis on shape per se. Later, in the mid- to late 1990s, some researchers revived interest in allometry and introduced the method that is currently most widely used for characterizing allometry in geometric morphometrics, the multivariate regression of shape on centroid size (Loy et al. 1996, 1998; Monteiro 1999).

The key advance of geometric morphometrics by comparison to earlier approaches is that it uses the complete information about a configuration of landmarks: not just a selected set of distances, but all aspects of the relative arrangement of the landmarks and all the interrelations among them. Whereas Mosimann (1970) characterized shape by the proportions of an object, geometric morphometrics considers all aspects of shape including proportions, angles, and the relative arrangement of parts. Formally, shape is defined as all the geometric features of a landmark configuration except for its size, position, and orientation (e.g., Dryden and Mardia 1998). Through this definition, geometric morphometrics offers a straightforward extension to the methods of the Gould–Mosimann school. Indeed, the majority of geometric morphometric studies can be seen as standing firmly in that tradition, particularly the analyses of allometry by multivariate regression of shape on centroid size (e.g., Monteiro 1999).

The separation of size and shape, however, has also been questioned (e.g., Richtsmeier and Lele 1993; O’Higgins and Milne 2013), and therefore, several authors have independently sought to develop methods for the analysis of the form of landmark configurations, encompassing size and shape together (Ziezold 1977; Kendall 1989; Dryden and Mardia 1992; Le 1995; Mitteroecker et al. 2004; Langlade et al. 2005; Goswami 2006a). The specifics of the methods differ, but overall, this type of analysis is in the spirit of the Huxley–Jolicoeur school. This insight is useful for understanding these morphometric methods and the associated procedures for characterizing allometry. Among these methods, the one that is the most widely known in geometric morphometrics is the size–shape space or Procrustes form space, which adds log-transformed centroid size as an extra dimension to the shape tangent space (Mitteroecker et al. 2004, 2013; Weber and Bookstein 2011). The other main approach, known under a variety of names such as size-and-shape space or allometric space, but also has been called form space, involves a superimposition of the landmark configurations without standardizing to unit centroid size (Ziezold 1977, 1994; Kendall 1989; Dryden and Mardia 1992; Le 1995; Langlade et al. 2005; Goswami 2006a). For these methods, as it is usual for methods within the Huxley–Jolicoeur school, allometry is characterized by finding a line of best fit in the multidimensional space (Mitteroecker et al. 2004, 2013).

The following three sections introduce and compare these methods, starting with the approach focusing on shape and its association with size, which has become very widespread in geometric morphometrics and is clearly allied to the Gould–Mosimann school, and then the two alternative methods that consider form, consisting of size and shape together, and thus are more or less squarely in the Huxley–Jolicoeur tradition. For each of the three methods, I present the respective morphological space as a starting point for the discussion.

As a guide through the confused terminology in this area, I have compiled the concepts and synonyms in Table 1, and after thorough consideration, I have decided to propose a new term for one of the concepts (“conformation” for “size-and-shape” and its various synonyms, none of which has been widely used in geometric morphometrics). Detailed explanations can be found in the respective section below.

**Table 1:** The different frameworks for allometry in geometric morphometrics, the schools of thought to which they belong, and some relevant concepts and the respective synonyms

	Shape	(Procrustes) form	Conformation
Definition	All geometric features of an object except for size, position, and orientation	The shape of an object combined with the log-transformed centroid size	All geometric features of an object except position and orientation
School	Gould–Mosimann	Huxley–Jolicoeur (with aspects of Gould–Mosimann school)	Huxley–Jolicoeur
Synonyms	None	Size–shape (Mitteroecker et al. 2004, 2005)	Size-and-shape (Kendall 1989; Dryden and Mardia 1992, 1998; Goodall and Mardia 1993; Le 1995; Kendall et al. 1999)
			Form (Goodall 1991)
			Figure (Ziezold 1977, 1994)
Space	Shape space	Form space (Mitteroecker and Gunz 2009; Weber and Bookstein 2011; Mitteroecker et al. 2013)	Conformation space
		Procrustes form space (Bastir et al. 2007; Mitteroecker and Gunz 2009; Mitteroecker et al. 2013)	
Synonyms for the space	None	Size–shape space (Mitteroecker et al. 2004, 2005)	Size-and-shape space (Kendall 1989; Dryden and Mardia 1992, 1998; Le 1995; Kendall et al. 1999)
			Form space (Goodall 1991; Rohlf 1996)
			Allometry space (Langlade et al. 2005)

## Allometry in shape space: regression of shape on size

The most widespread method for studying allometry in geometric morphometrics is multivariate regression of shape on centroid size or log-transformed centroid size (e.g., Monteiro 1999; Rosas and Bastir 2002; Drake and Klingenberg 2008; Rodríguez-Mendoza et al. 2011; Weisensee and Jantz 2011; Klingenberg et al. 2012; Ponssa and Candioti 2012; Mitteroecker et al. 2013; Murta-Fonseca and Fernandes 2016). Because size and shape are logically separate, the multivariate regression analysis can test whether there is a statistical association between them and, if so, provides a characterization of the allometry as the expected shape change per unit of increase in the size variable. This is a direct implementation of the Gould–Mosimann framework of allometry for geometric morphometrics.

### Shape spaces

For understanding this approach and its relation to the other methods discussed in this paper, it is helpful to consider how size and shape are quantified (Dryden and Mardia 1998). Centroid size is the measure of size used almost universally in geometric morphometrics: it is the square root of the sum of squared distances of all the landmarks of an object from their centroid (center of gravity, whose location is obtained by averaging the *x* and *y* coordinates of all landmarks). Centroid size fulfills Mosimann’s (1970) conditions for a standard size variable. To quantify the shape difference between two landmark configurations, Procrustes superimposition can be used: both configurations are scaled to have centroid size 1.0 and are transposed and rotated so that the sum of squared distances between corresponding landmarks is minimal (this involves a translation so that both configurations share the same centroid). The square root of the sum of squared distances between corresponding landmarks is called Procrustes distance: it is the discrepancy between the landmark configurations that cannot be removed by scaling, translation, or rotation and is therefore useful as a measure of shape difference.

**Fig. 5:**
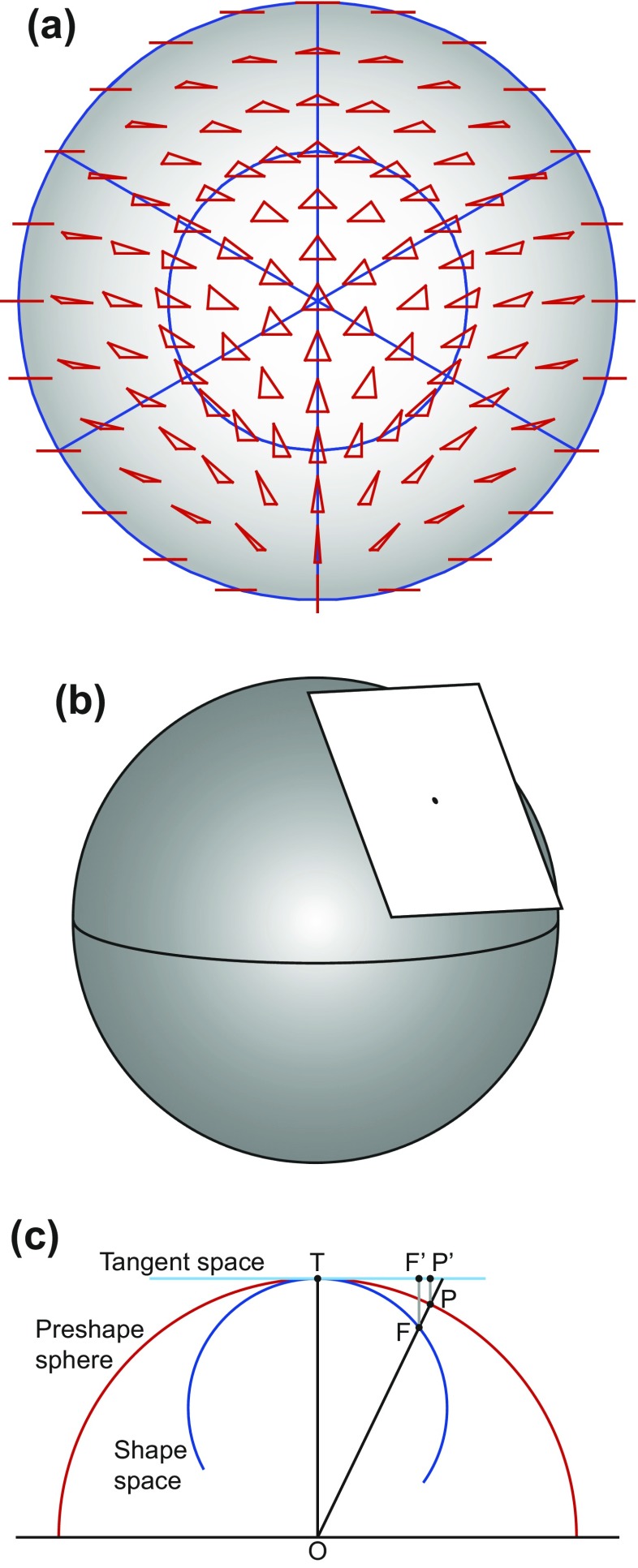
Kendall’s shape space for triangles. **a** A view of one half of the shape space for triangles with some examples shown in the respective locations. The “pole” corresponds to an *equilateral triangle*. The equator contains the *flat triangles* where all three vertices are on a straight line. The six “meridians” correspond to *isosceles triangles*. The hidden hemisphere of the shape space contains mirror images of the triangles visible in this diagram. **b** The tangent space, a plane in this case, touching the sphere of the shape space at an arbitrary location. **c** The connection between Procrustes superimposition and Kendall’s shape space for triangles (modified after Rohlf 1999). The preshape sphere and tangent space are aligned to the shape space so that all three touch at point T, the point that corresponds to the mean shape in the sample. The shape space is only shown in part to emphasize that the alignment with the preshape sphere is only valid in the neighborhood of point T

Kendall’s shape space is a representation of all possible shapes with a given number of landmarks and a given dimensionality (i.e., coordinates measured in two or three dimensions), so that the distance between the points representing any two shapes corresponds to the Procrustes distance between the respective shapes (Kendall 1984; Small 1996; Dryden and Mardia 1998; Kendall et al. 1999). These shape spaces are complex, multidimensional analogs of curved surfaces and are therefore difficult to visualize for all but the simplest landmark configurations. A shape space that can be visualized with relative ease is the one for triangles in two dimensions, which turns out to be the surface of a sphere (Fig. 5a). On this sphere, every possible triangle shape has its particular place (the only exception is the totally degenerate triangle whose vertices are all exactly in the same point, but one can reasonably question whether this really is a triangle shape at all). A helpful way to appreciate the arrangement of shapes is to orient the shape space so that an equilateral triangle is at the “north pole” (its antipode, which is its mirror image and thus also an equilateral triangle, then is the “south pole”). In this orientation, the “equator” contains the collinear triangles, where all three vertices are aligned along a straight line, and there are six “meridians” that contain isosceles triangles (Fig. 5a). These particular properties are specific to the shape space for triangles in two dimensions, but a general feature of Kendall’s shape spaces is that every possible shape has its specific place in the shape space with the relevant numbers of landmarks and dimensions. Every point in a shape space corresponds to a shape, and every shift from one point to another corresponds to a shape change (the direction of the shift corresponds to a class of shape changes that are the same except for their magnitude). There is a shape space for any number of landmarks and any number of dimensions (Kendall et al. 1999), although geometric morphometrics is mostly concerned with shapes in two or three dimensions (perhaps one dimension in specific cases). Also, these shape spaces exist without depending on any particular samples.

Because the shape spaces are complex, multidimensional equivalents of curved surfaces, they are not amenable to standard statistical analyses. But just as a flat map can be a good approximation of the surface of the earth in a limited region, perhaps up to several hundred kilometers across, it is possible to use a linear tangent space to approximate the shape space in the neighborhood of the point where the two spaces touch (Fig. 5b; Goodall 1991; Dryden and Mardia 1998; Rohlf 1999). Within the tangent space, standard multivariate analyses can be used. But just as for the maps of the earth, distortions appear if the shape changes represented in the tangent space are large (think of the distortions to Greenland and Antarctica in many world maps). By choosing the mean shape in the sample as the tangent point, it is possible to minimize this distortion. Fortunately, in most biological datasets, shape variation is sufficiently limited for such distortions to be negligible, even in studies that include a substantial range of shapes such as skull shapes across the orders of mammals (Marcus et al. 2000).

In practice, a local approximation of the tangent space for a dataset can be obtained from a generalized Procrustes superimposition of the landmark configurations (Goodall 1991; Dryden and Mardia 1998). First, all the landmark configurations are scaled to unit centroid size by dividing all the landmark coordinates by the centroid size of the respective configuration. Variation in position is removed by centering the configurations, so that they all share the same centroid, at the origin of the coordinate system. These configurations, standardized for size and position, are called preshapes (Goodall 1991; Dryden and Mardia 1998). Finally, variation in the orientation of the landmark configurations is removed in an iterative procedure. Initially, an arbitrary configuration (usually the first in the dataset) is taken and all other configurations are aligned to it to minimize the sum of squared distances between corresponding landmarks. The landmark coordinates of all configurations (including the one to which all others were aligned) are then averaged and rescaled to have unit centroid size. Then this consensus configuration is used as the target and all landmark configurations are aligned to it. This procedure of aligning and averaging is repeated until the sum of squared distances between corresponding landmarks no longer changes (or equivalently, until the consensus no longer changes). This usually takes only very few iterations (two or three are sufficient in many datasets). As a result of this procedure, the aligned preshapes are arranged on a portion of a sphere of unit radius (due to the standardization of centroid size) surrounding the consensus (red half-circle in Fig. 5c). For a limited range of shapes, this arrangement of preshapes is a local approximation of the arrangement of the corresponding shapes in Kendall’s shape space, and from either of them, the projection onto the tangent space yields a further local approximation. Therefore, the Procrustes superimposition and projection to tangent space can provide a local, linear approximation of the arrangement in shape space. This is true even for landmark configurations with many landmarks, where shape spaces are complex and have very many dimensions so that they cannot be visualized as in Fig. 5. In this case, using multivariate analyses in the tangent space is the most feasible option for exploring the local structure of the shape space in the vicinity of the average shape.

### Multivariate regression

For studying allometry with geometric morphometric data, the most straightforward method is to use a multivariate regression of the shape variables onto a measure of size (Monteiro 1999). The shape variables are the coordinates of the specimens in the shape tangent space and the shape measure is centroid size or the logarithm of centroid size. Because the scaling step of the Procrustes superimposition removes variation of size, the shape tangent space does not contain any size variation, and therefore size and shape are logically separate from each other. Thus, it makes sense to test whether there is allometry by examining whether size and shape are correlated statistically. If so, the patterns of allometry can be characterized as the expected shape change per unit of increase in size. Multivariate regression and the tests associated with it can perform both those tasks (Monteiro 1999). This regression-based approach is completely in the spirit of the conceptual framework of the Gould–Mosimann school. It characterizes allometric variation of shape as a consequence of variation in size, which clearly reflects Gould’s (1966) definition of allometry as “the study of size and its consequences” (p. 587). Also, the concepts of size and shape used in geometric morphometrics are directly compatible with those of Mosimann (1970), and the regression method and associated statistical tests are closely related to the multiple correlation methods used by Mosimann.

Multivariate regression uses several dependent variables and one or more independent variables (Mardia et al. 1979; Johnson and Wichern 1988; Timm 2002). It should not be confused with multiple regression, where there is one dependent variable and several independent variables (if there are several independent variables in a multivariate regression, it is sometimes called multivariate multiple regression). For allometry, the multivariate regression model can be written as follows: **y** = **c** + **b***x* + **ε**, where **y** is the random vector of shape (as tangent space coordinates), **c** is a constant vector analogous to an intercept, **b** is the vector of regression coefficients, *x* is centroid size (or log-transformed centroid size), and **ε** is the error term. Because the coordinates in tangent space are centered at the mean shape, the intercept term can be dropped if size is expressed as the deviation from the average size in the sample. Therefore, the main quantity of interest is the vector of regression coefficients, which indicates the shape change expected per unit of increase in centroid size. This vector can be obtained as **b** = cov(*x*, **y**)/var(*x*). This is simply a vector of bivariate regression coefficients of the shape variables on centroid size (or log-transformed centroid size). In the context of geometric morphometrics, this regression vector can be visualized directly as a shape change (Monteiro 1999; Rosas and Bastir 2002; Rodríguez-Mendoza et al. 2011; Klingenberg 2013b).

For testing the statistical significance of the association between size and shape, there are different methods available: parametric tests or nonparametric permutation tests, and for each of these, there is a choice between the classical multivariate test statistics (e.g., Wilks lambda, Pillai’s trace, or Roy’s maximum root; e.g., Timm 2002) or a test statistic based on Goodall’s (1991) *F* statistic. Goodall’s *F* statistic is calculated by adding up sums of squares across all coordinates and all landmarks (for total, predicted, and residual components of variation) and computing the appropriate ratio of the resulting sums. The approach based on Goodall’s *F* statistic is widely used because it tends to show good performance even with relatively small sample sizes. In the context of allometric regression, Goodall’s *F* has been used as part of parametric tests (Monteiro 1999). As an alternative, there are permutation tests that implement the null hypothesis of independence between size and shape by randomly reassociating shapes and sizes among specimens (Pitman 1937; Good 2000). Permutation tests have the advantage that they do not make any assumptions about the particular distribution of the data, which is why they have been implemented in standard morphometrics software and are widely used (e.g., Drake and Klingenberg 2008; Rodríguez-Mendoza et al. 2011; Weisensee and Jantz 2011; Klingenberg et al. 2012; Martín-Serra et al. 2014). The predicted sum of squares, as a proportion or percentage of the total sum of squares, is an intuitive indication for how much of the shape variation the regression can account.

To assess visually how well the data fit a straight-line relationship, it is desirable to have plots as in bivariate regression, but it is not immediately clear what shape variable should be plotted against size. Early studies used plots of Procrustes residuals for individual landmarks (Walker 1993) or principal component scores (Loy et al. 1998; O’Higgins and Jones 1998; Birch 1999) against centroid size or another size measure. These plots are not optimal for various reasons, especially when factors other than size also have effects on shape variation (e.g., different taxa, sex dimorphism, phenotypic plasticity). A better option is to compute regression scores by projecting the data points in shape space onto an axis in the direction of the regression vector (Drake and Klingenberg 2008). This is the shape variable that has the maximal covariation with centroid size (or log-transformed centroid size, if that was used as the independent variable) and is therefore an optimal summary variable. Plots of regression scores versus centroid size show both the predicted component of shape variation and that part of the residual variation that is in the direction of the regression vector. The plots therefore can give a visual impression how closely the data points fit a straight line.

**Fig. 6:**
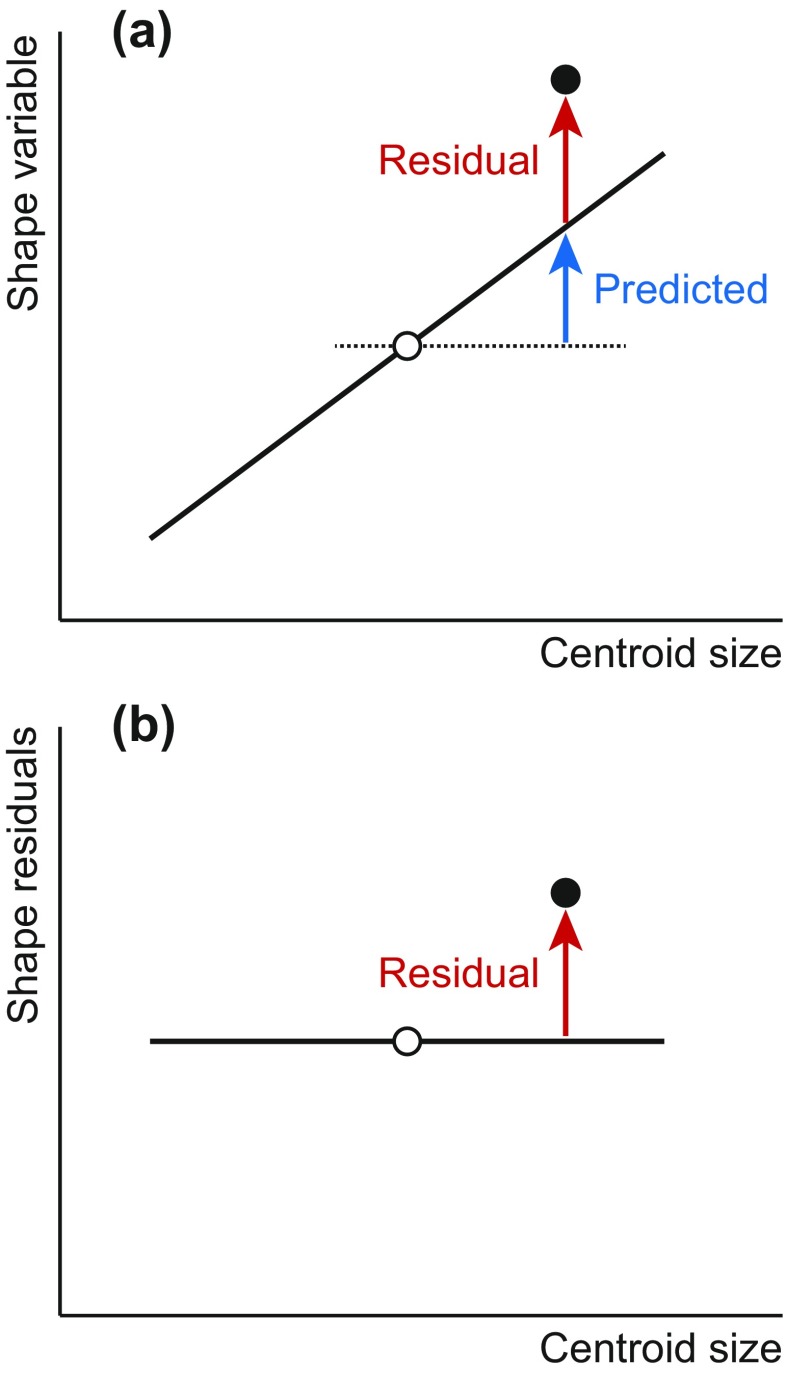
Allometric regression and size correction. **a** The decomposition of the shape deviations of each data point (*black dot*) from the sample average (*hollow dot*) into predicted and residual components. For each shape variable, the predicted component can be computed from the deviation in size of the specimen of interest from the average size in the sample and the slope of the regression line (*solid line*). **b** Size correction by using residuals from the regression. For the residual component of variation, there is the same expected value for shape regardless of the size of a specimen (*horizontal line*)

The regression partitions the total variation of each dependent variable into a component of variation that is predicted by the independent variable(s) and a residual component that is variation for which the regression cannot account (the “error” component in the regression model). These components are computed for each dependent variable separately (Fig. 6a). In the context of analyses of allometry, the dependent variables are shape variables (usually Procrustes coordinates or perhaps, and equivalently, partial warp scores) and the sums of squared deviations for the total, predicted, and residual components can be added up across variables as Procrustes sums of squares. The predicted and residual components can also be expressed as a percentage of the total variation, which is a useful and intuitive way to quantify the relative importance of allometry for the shape variation in a dataset. Many studies of allometry have used this approach in different contexts and often found that allometry accounts for small to moderate proportions of the total shape variation (Drake and Klingenberg 2008; White 2009; Rodríguez-Mendoza et al. 2011; Weisensee and Jantz 2011; Klingenberg and Marugán-Lobón 2013; Mitteroecker et al. 2013; Sherratt et al. 2014; Golubović et al. 2015; Viscosi 2015), but this proportion can reach 30 % or even 50 % in some instances (Klingenberg et al. 2012; Openshaw and Keogh 2014; Murta-Fonseca and Fernandes 2016; Strelin et al. 2016).

There are some practical points that are useful to consider for studies of allometry using multivariate regression of shape on size. Overall, multivariate regression is statistically well behaved and, when tests use statistics based on Procrustes sums of squares and Goodall’s *F*, has considerable statistical power even with fairly small sample sizes. There are some limitations imposed by the data, however, which can lead to unstable estimates of allometry. Estimates are unstable if the relationship between shape and size is weak and also if the sample only includes a limited range of sizes. The strength of the allometric relation is usually not under the control of the investigator (it is one of the aspects that is under study), but the range of sizes is a question of the study design. If only a small range of sizes is available, so that only a short section of the allometric trajectory is covered by the data, the proportion of shape for which allometry accounts will be underestimated and the estimated patterns of allometric shape changes may differ considerably from the true allometric patterns. It is helpful to try to include the extremes of the size distribution, the smallest and largest specimens that are available, because these specimens can make a substantial contribution to stable estimates of allometry. If the range of sizes is very large, it is often useful to use log-transformed centroid size as the independent variable in the multivariate regression (Zelditch et al. 2000; Mitteroecker et al. 2004, 2013; Klingenberg et al. 2012; Klingenberg and Marugán-Lobón 2013). The effect of the log-transformation can be likened to stretching the scale of size for small values and shrinking it for large values—because much of the allometric change is usually concentrated among the smaller sizes, this often results in a better fit to a straight-line relationship.

Although multivariate regression of shape on size is the most widespread method for characterizing allometry in geometric morphometrics, some authors have used different methods. In particular, many studies used bivariate regression or correlation of the PC1 of shape versus centroid size (or log-transformed centroid size) for testing and displaying allometry (O’Higgins and Jones 1998; Singleton 2002; Zollikofer and Ponce de León 2002; Kölliker-Ott et al. 2003; Sardi et al. 2007; Morimoto et al. 2008; Sardi and Ramírez Rozzi 2012; Watanabe and Slice 2014). This method has some drawbacks because, unless size is the only factor contributing appreciably to morphological variation, there is no reason why the PC1 or any other PC necessarily is associated with allometry. Allometric shape changes, even if allometry is perfectly linear in shape space, may be in a direction of shape space that is at oblique angles to the first few or even to all PCs. This is the reason why some studies have found that more than one PC or PCs other than the PC1 are correlated with centroid size (Singleton 2002; Cobb and O’Higgins 2007; Sardi et al. 2007; Weisbecker 2012; Watanabe and Slice 2014) and no PC of shape can be expected to provide a complete or optimal characterization of allometry. By contrast, if the allometric trajectory is linear in shape space, multivariate regression of shape on size is guaranteed to provide an optimal estimate (and it is likely to provide a reasonable approximation even if there is moderate nonlinearity). The regression approach is therefore the preferred method for analyzing allometry within the Gould–Mosimann framework.

### Analysis of allometry in multiple groups

Many datasets contain multiple groups, such as specimens of different species, sexes or from different locations. Such group structure needs to be taken into account both because it can pose a number of statistical difficulties and because, in many instances, it offers opportunities for inferring the biological processes responsible for the observed variation. For instance, it can be useful to compare within- and among-group allometry (Gonzalez et al. 2011; Klingenberg et al. 2012; Klingenberg 2014) or the allometries of different groups can be compared to each other (Mitteroecker et al. 2004; Adams and Nistri 2010; Rodríguez-Mendoza et al. 2011; Cardini and Polly 2013; Lazić et al. 2015).

**Fig. 7:**
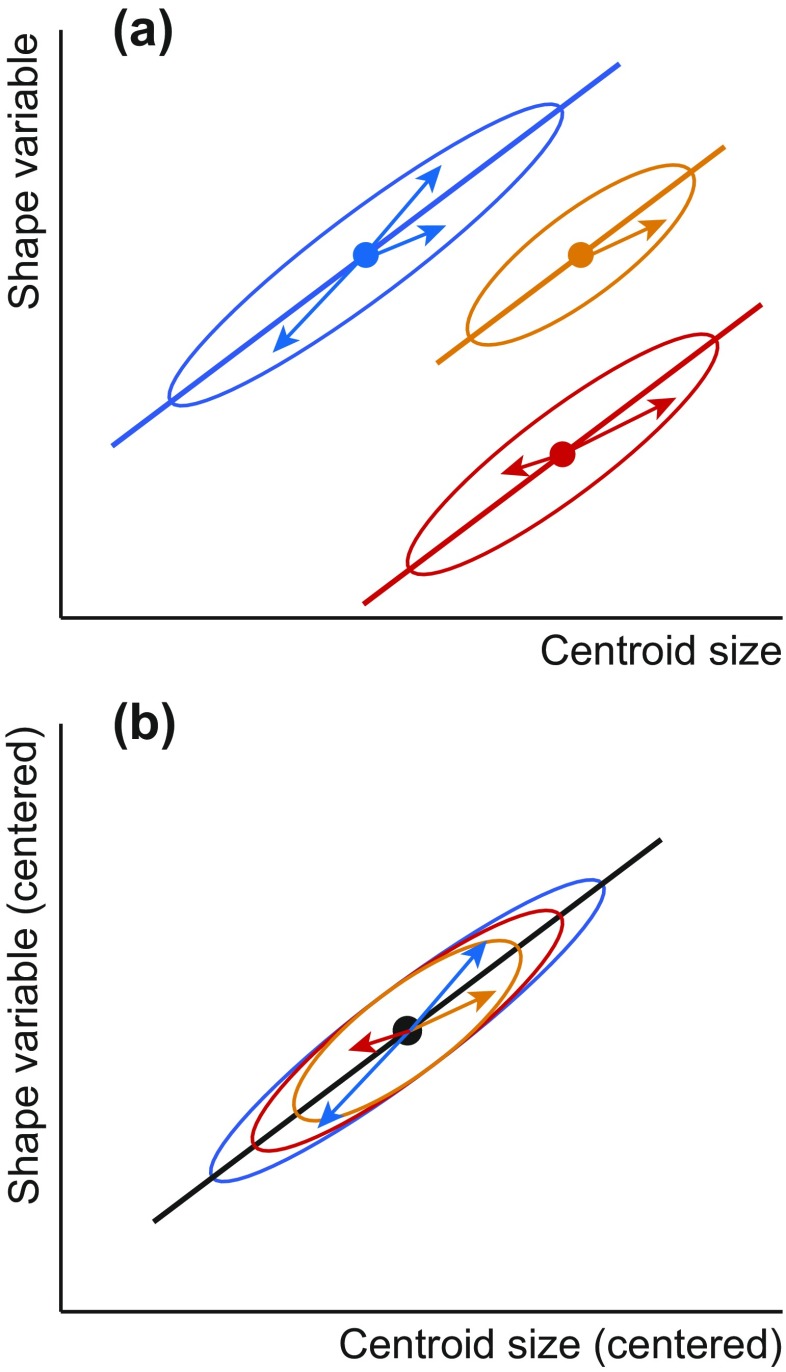
Pooled within-group regression. **a** A plot of uncentered variables. All three groups share the same regression slope, as assumed by the method. Computations are based on the deviations of the data points from the respective group averages (*arrows*), rather than deviations from the grand average. **b** A plot of group-centered variables. The scatters of the different groups have been moved to share the same shape and size averages. After this centering step, an ordinary regression analysis provides a joint estimate of the shared within-group allometry

In many situations, a common estimate for the allometry in several groups is required, for instance when a simultaneous estimate of the allometry within several groups is to be contrasted to the among-group allometry, for the study of ontogenetic scaling, or for size correction in taxonomic studies (Frost et al. 2003; Mitteroecker et al. 2004; Gonzalez et al. 2011; Sidlauskas et al. 2011; Klingenberg et al. 2012; Strelin et al. 2016). A method that can achieve such an estimate is pooled within-group regression, which is equivalent to multivariate analysis of covariance (MANCOVA), a long established method in multivariate statistics (e.g., Timm 2002). Pooled within-group regression uses the shape and size deviations of each specimen from the shape and size averages of the group to which that specimen belongs, not the grand mean, to compute variances and covariances (Fig. 7a). Equivalently, pooled within-group regression can be explained as a two-step procedure where the differences among group averages are first removed by centering the shape and size data by group and then an ordinary regression is carried out on these centered data (Fig. 7b). To visualize how well the data fit a straight-line relation, it is possible to use regression scores, which, in the context of pooled within-group regression, have been called the “common allometric component” (Mitteroecker et al. 2004). The assumption that underlies the method is that all groups share the same allometry (the regression coefficients are the same across groups). If the groups differ in the range of size variation (as in Fig. 7), the groups with a greater range of sizes have a greater effect on the regression estimates. As long as the assumption of equal regression coefficients holds, this property leads to an optimal estimate because groups with a greater range of size variation contain more information on allometry (groups with very little size variation, which produce unreliable estimates of allometry, have only very little weight in the joint estimation of within-group allometry).

A key assumption in pooled within-group regression is that all the groups share a common allometry, that is, that the regression coefficients are the same across all groups. This assumption justifies the calculation of a single estimate of the allometric pattern within groups. In principle, it can be tested using the statistical tests available as part of the MANCOVA methods (Zelditch et al. 2000; Frost et al. 2003; Zelditch et al. 2003; Ljubisavljević et al. 2010; Viscosi and Cardini 2011). Yet, the statistical significance from these tests is problematic as a criterion for deciding whether or not a pooled within-group regression should be used. If there is any difference between the regression vectors of two groups, no matter how small it is, increasing sample sizes will always produce a significant result at some point. This leads to the perverse situation that an investigator with a bigger dataset cannot conduct the analyses that are permissible with a smaller dataset. This can be serious for a wide range of applications, including size correction for taxonomic purposes or the separation of allometric and nonallometric components of sex dimorphism (Gidaszewski et al. 2009; Sidlauskas et al. 2011). A solution is to relax the conditions for using pooled within-group regression by adopting a more flexible version of the justification for pooled within-group regression: it provides a simultaneous estimate of a common allometric pattern if all groups do indeed share the same allometry, or it provides a compromise estimate as long as the allometric patterns in all the groups are sufficiently similar for such a compromise to be sensible. This criterion requires some judgment by the investigator, and above all, the magnitude of the differences among the allometries of different groups needs to be taken into account. Small angles or very high vector correlations between the regression vectors of different groups can be taken as indications that differences are negligible and therefore that a compromise across groups is justifiable.

Comparing the allometries in different groups is another important task in the analysis of allometry (Klingenberg 1996b). A wide range of methods for comparing allometric patterns have been used in traditional morphometrics, such as computing the angles between allometric trajectories (Boitard et al. 1982; Cheverud 1982; Gibson et al. 1984; Klingenberg and Zimmermann 1992a; Wilson and Sánchez-Villagra 2010; Wilson 2013) or generating an ordination of the allometric patterns by a PCA of the allometric vectors of different groups (Solignac et al. 1990; Klingenberg and Froese 1991; Klingenberg and Spence 1993; Gerber et al. 2008; Wilson and Sánchez-Villagra 2010; Wilson 2013). With some minor adaptations, these approaches can also be used in the context of geometric morphometrics. Angular comparisons between allometric trajectories estimated by multivariate regressions of shape on size have been used in a number of studies (Zelditch et al. 2000, 2003; Frédérich et al. 2008; Gonzalez et al. 2010, 2011; Frédérich and Vandewalle 2011; Urošević et al. 2013). Gonzalez et al. (2010) summarized a number of such comparisons graphically as a dendrogram obtained from angles between allometric vectors using the UPGMA clustering method. Frédérich and Vandewalle (2011) and Urošević et al. (2013) conducted ordination analyses of allometric trajectories by nonmetric multidimensional scaling of the matrix of pairwise angles between allometric vectors. There is clearly potential for further work in this area.

### Size correction

An important application of allometry is size correction. Although extracting the shape information from the raw data of landmark coordinates removes variation in size per se, but the shape data may still contain a component of size-related shape variation due to the effects of allometry. Such allometric shape variation can influence taxonomic studies or analyses of morphological integration and modularity (Klingenberg et al. 2003; Mitteroecker and Bookstein 2007; Klingenberg 2009, 2013a; Sidlauskas et al. 2011). The multivariate regression approach offers a logical and straightforward method to identify and possibly remove the allometric component of shape variation.

The multivariate regression provides a useful means for size correction as it partitions the variation in the dependent variables into predicted and residual components (Fig. 6). The predicted component corresponds to allometric variation of shape, whereas the residual component encompasses the nonallometric variation. The residuals are uncorrelated with the size measure used as the independent variable in the regression (usually centroid size or log-transformed centroid size). Furthermore, if the assumption of a linear relation between size and shape is met, the expected value for the residuals is the same for every size (Fig. 6b). For these reasons, the residuals from a multivariate regression of shape on size are the optimal choice for size correction in the context of the Gould–Mosimann framework of allometry.

Note that the dimensionality of the shape data after size correction using the regression approach is usually the same as before. Allometric effects of size usually do not account for all the variation in any direction of shape space, and so there tends to be some variation left in every possible shape variable. This is markedly different from the analyses in the tradition of the Huxley–Jolicoeur framework, where allometric and nonallometric components of variation are orthogonal in the phenotypic space. The difference arises from the fact that size is extrinsic to the shape space. Within the shape space, factors other than size can also contribute to variation of the shape feature that corresponds to the allometric vector. As a result of this, plots of size versus the regression score, the projection of the data points onto the direction of the allometric vector (Drake and Klingenberg 2008), usually do not fit perfectly to a straight line.

Size correction by using residuals from multivariate regression of shape on size is widely used in morphometric studies. Because the residuals are in the same coordinate system as the original shape data, just with the predicted component of shape variation removed, the size-corrected data can be used by any morphometric technique for further analyses. A particular focus for such applications is the study of morphological integration and modularity, where accounting for allometric effects is especially important because allometry is a known factor contributing to integration (Klingenberg et al. 2001, 2003; Klingenberg 2009; Ivanović and Kalezić 2010; Martínez-Abadías et al. 2011; Jojić et al. 2012; Klingenberg and Marugán-Lobón 2013; Barbeito-Andrés et al. 2015).

**Fig. 8:**
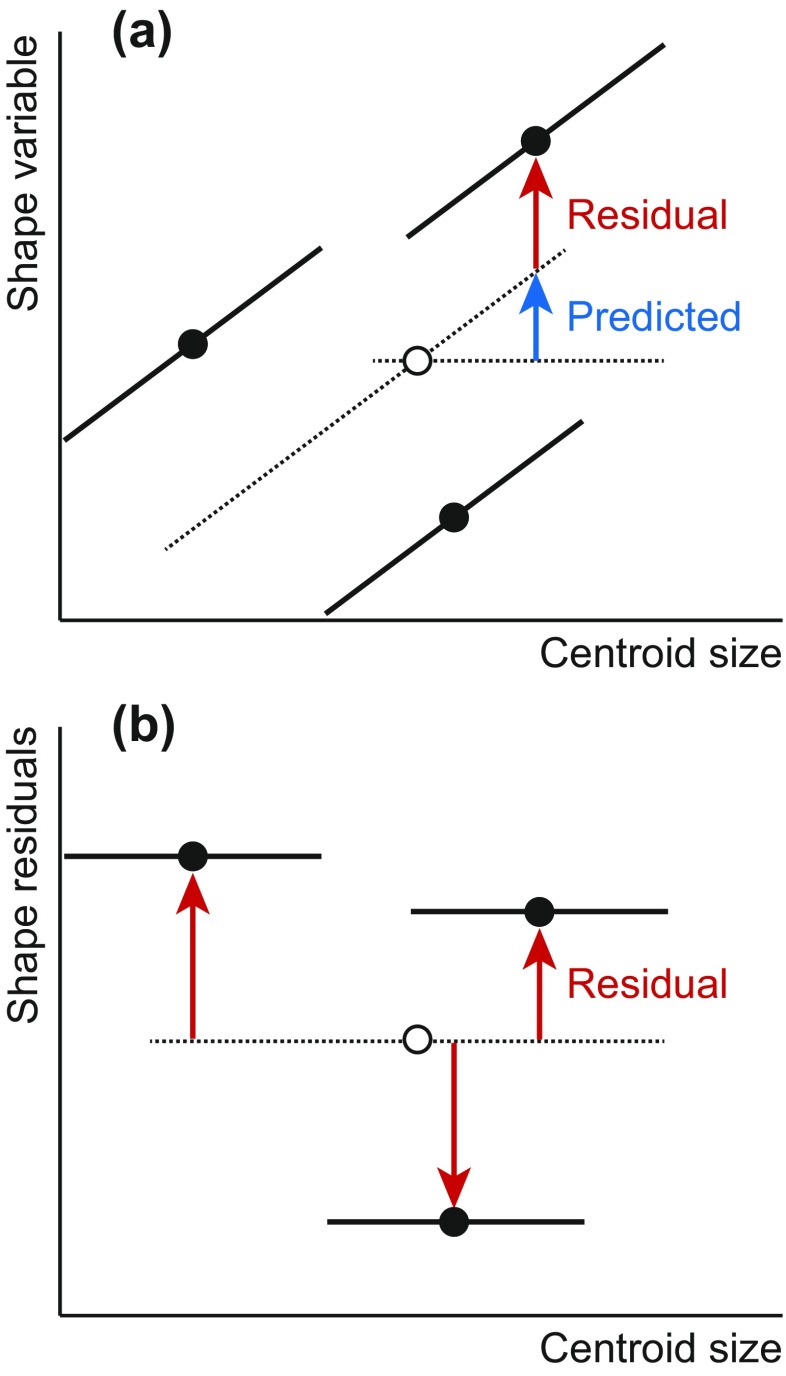
Allometric regression and size correction for multiple groups. **a** Computation of the predicted and residual components of variation. If deviations from the grand mean for size and shape (*hollow dot*) and the slope from a pooled within-group regression of shape on size are used, group differences are partitioned into the correct predicted and residual components. **b** Size correction using residuals from pooled within-group regression. The shape residuals are uncorrelated to size within groups, but overall, there may be correlations with size depending on the size and shape differences among groups

Many studies use size correction in a situation where the data are from multiple groups such as sexes or species (Rosas and Bastir 2002; Mitteroecker et al. 2004; Gidaszewski et al. 2009; Sidlauskas et al. 2011; Strelin et al. 2016). A pooled within-group regression of shape on size can take this kind group structure into account. Further, in this case, it is important to apply the decomposition of variation into predicted and residual components of variation not only to the variation within groups, but also to the differences between them. Only in this way is it possible to determine how much of the difference between groups is due to allometry. By computing predicted values and residuals based on deviations from the grand mean, rather than from the various group means (Fig. 8a), this decomposition of group differences into predicted and residual components can be achieved easily. The resulting residuals are uncorrelated to size within groups, provided that the assumptions of the pooled within-group regression are met, although there may be correlations between the group averages and size (Fig. 8b). Discrimination between groups is often improved after such a size correction, which is why it can be a very useful tool in taxonomic studies (Sidlauskas et al. 2011).

## Allometry in Procrustes form space

In recent years, several other approaches have been proposed that aim at characterizing morphological form by combining size and shape in a single space. One of these methods adds natural log-transformed centroid size as an extra dimension to shape tangent space to produce a “size–shape space” (Mitteroecker et al. 2004), also called “form space” or “Procrustes form space” (Bastir et al. 2007; Mitteroecker and Gunz 2009; Weber and Bookstein 2011; Mitteroecker et al. 2013).

**Fig. 9:**
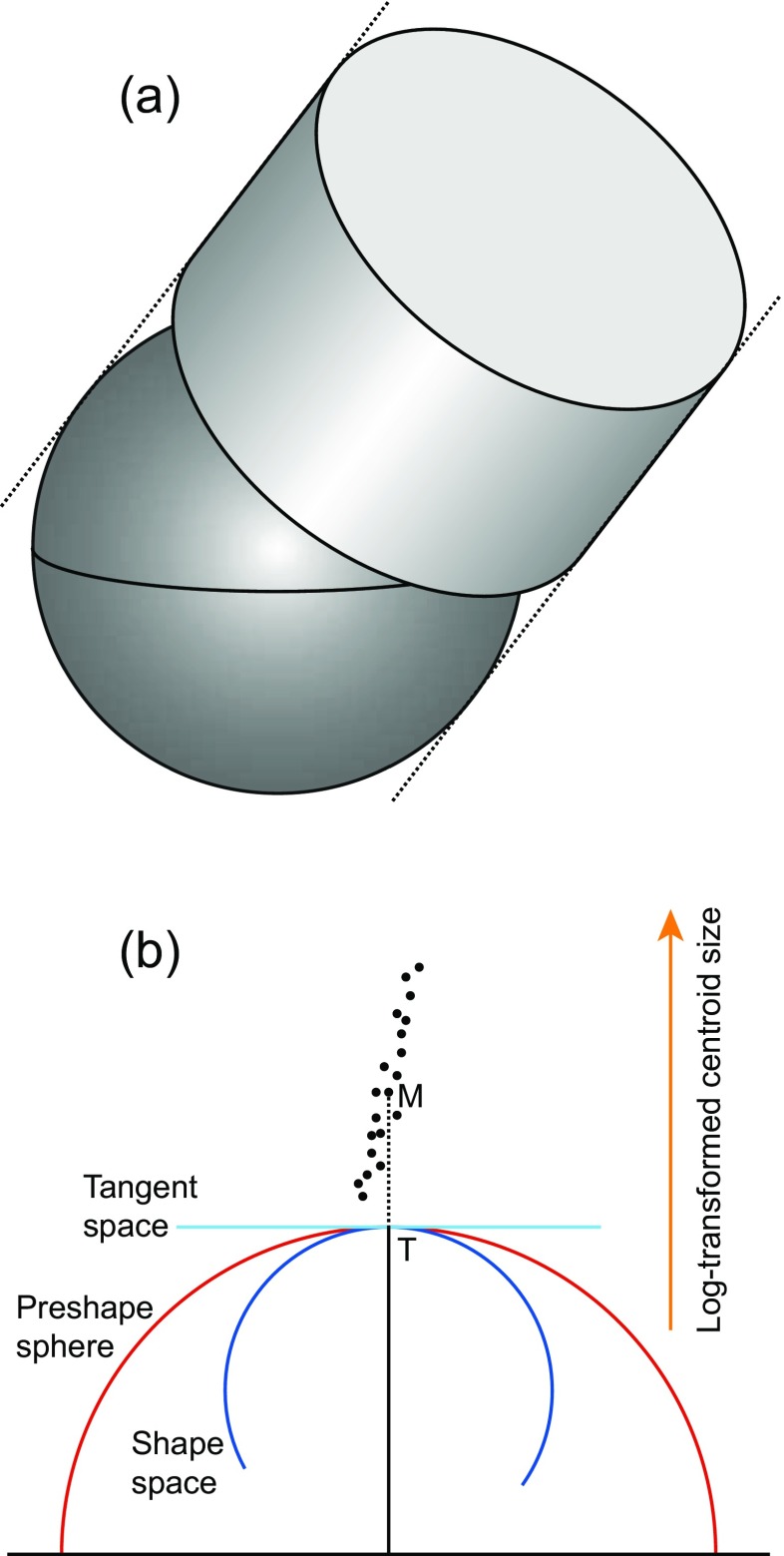
Procrustes form space. **a** The relation of the Procrustes form space to Kendall’s shape space for triangles. The Procrustes form space is shown in part, cut transversely through the plane of the shape tangent space to show Kendall’s shape sphere and through a parallel plane at the top of the diagram. The entire Procrustes form space is a cylinder that completely envelops the shape space and extends without limit in both directions along the axis of log-transformed centroid size (*dotted lines*). **b** Schematic cross-section of the Procrustes form space, shape tangent space, shape space, and sphere of aligned preshapes for triangles. The *black dots* indicate data points as they might occur in a biological dataset: size variation is clearly exceeding the shape variation. The shape corresponding to the mean form (M) is the same as the mean shape in the sample (at the tangent point T). There is a slight tendency for the data points to be further to the right with increasing values of log-centroid size, indicating allometry

Given the amount of attention that the structure of Kendall’s shape space has received in geometric morphometrics, it is somewhat surprising that there has been no discussion on the global structure of the Procrustes form space. Because the form space is a shape tangent space augmented by log-transformed centroid size as an additional dimension, the tangent space confers to it a strong relation to Kendall’s shape space (Fig. 9). The tangent space touches Kendall’s shape space at the location of the mean shape. The line from the center of the shape space to the location of the mean shape is by definition perpendicular to the tangent space and is also the main axis of the form space. In principle, there is no upper or lower limit for log-transformed centroid size and the form space can therefore extend in both directions along this axis without limit. The geometry of the cross section of the form space corresponds to the projection of the shape space onto the tangent space. For the shape space of triangles, this is a circle, so that the overall structure of form spaces for triangles is that of a cylinder (Fig. 9a). The diameter of the cylinder can be debated, depending on whether the projection to the tangent space is from the shape space (corresponding to a full Procrustes fit) or from the sphere of aligned preshapes (corresponding to a partial Procrustes fit). In practice, the details of the boundaries of the form space matter little, because both shapes and centroid sizes have quite limited ranges in actual biological data (Fig. 9b): size ranges very rarely cover more than two or three orders of magnitude, usually much less, and even comparisons at very large phylogenetic scales tend to occupy only quite small regions of the total shape spaces (Marcus et al. 2000; Sallan and Friedman 2012; Klingenberg and Marugán-Lobón 2013).

Because each Procrustes form space depends on a tangent space, its optimal properties are limited to landmark configurations with shapes that are reasonably close to the shape at the tangent point. For configurations with very different shapes, the distortions of the tangent projection will result in corresponding distortions in the Procrustes form space. In this way, Procrustes form spaces are fundamentally different from Kendall’s shape or size-and-shape spaces (Kendall 1989; Kendall et al. 1999), which can accommodate all configurations with the same number of landmarks and in the same dimensionality, no matter how different the arrangement of the landmarks may be.

Why is log-transformed centroid size used as the extra dimension to add to the shape tangent space for constructing the Procrustes form space? This question, about the scaling of the size axis relative to the other axes of the form space, is important because it can affect all further analyses performed in that space. The justification relies on the null model that the data are generated by isotropic variation of landmark positions around an average configuration, which means that variation is equal in every direction and at every landmark, and that the scale of variation is small (the deviations of landmarks from the respective average positions are small relative to the distances among landmarks in the average configuration). Under this model, it can be shown that natural log-transformed centroid size is on the same scale as the variables of the shape tangent space (Mitteroecker et al. 2004, Appendix). The isotropic null model, however, is biologically highly unrealistic and the question therefore arises whether natural log-transformed centroid size is still scaling properly in relation to the shape variables when the assumption of isotropic variation is abandoned. This question is not easily answered, because the condition that variation is small and isotropic plays a key role in the justification for using the natural logarithm transformation of centroid size. This caveat, whether the scaling of log-centroid size against the remaining dimensions of Procrustes form space is really appropriate for biological data that deviate strongly from the isotropic model, has been raised before (Cardini and Polly 2013; O’Higgins and Milne 2013). The log-transformation as such is likely to be sensible in many circumstances—if there are large amounts size variation, it will compensate for the tendency that most shape changes occur at relatively small sizes for most organisms and, if there is very little shape variation, the log-transformation will not make much of a difference. The choice of the basis for the logarithms, and thus the relative scaling of variation in log-centroid size versus the variation in shape, is more difficult to justify without referring to the isotropic model. Some results of analyses in Procrustes form space are more affected by this possible uncertainty than others.

**Fig. 10:**
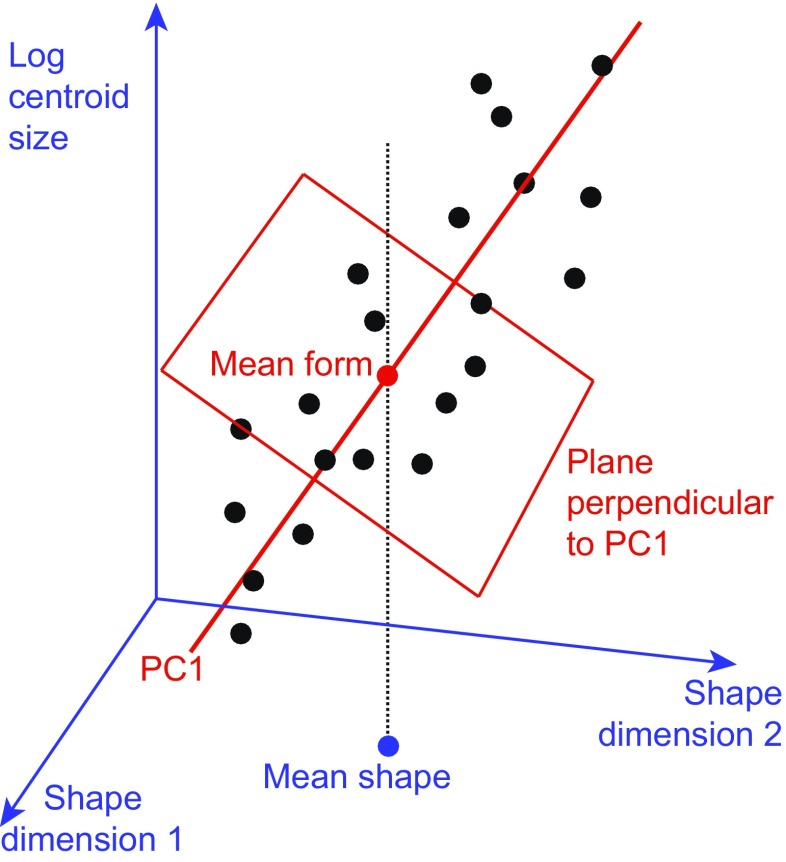
Analysis of allometry in Procrustes form space. This is a close-up view of just that part of the Procrustes form space that is actually occupied by data points (cf. Fig. 9b). If there is allometry, the PC1 is inclined relative to the axis of log-centroid size. As a consequence, the plane perpendicular to the PC1 is also inclined, which implies that some variation for log-centroid size remains in the form data even after size correction

Because Procrustes form space includes log-transformed centroid size, the regression approach used to characterize allometry in shape space is not applicable, nor is the general logic of the Gould–Mosimann school that defines allometry as an association between size and shape. Instead, the analysis of allometry needs to follow the tradition of the Huxley–Jolicoeur school, which focuses on the covariation among variables within a morphological space and attempts to find a line of best fit to the data as an estimate of the allometric trajectory. Because the variation of log-transformed centroid size is far greater than for any of the shape variables in most morphometric datasets, the PC1 of the Procrustes form data usually is associated closely with the axis of log-centroid size. If there is allometry in the data, this means that there is a tendency for shape to change with increasing size, which implies that the PC1 is slightly inclined relative to the axis of log-centroid size (Fig. 10). Note that, as for traditional morphometric data, the approach using the PC1 in form space is based on the assumption that variation in size or related to it is dominant over variation from other origins. This is often true in homogeneous samples and especially if the sample contains ontogenetic variation. If the data are structured by other factors, however, such as different species, sexes, or ecomorphs, the PC1 may fail to characterize allometry properly.

The opposite to allometry is isometry, and this is therefore a possible null hypothesis that can be used in statistical tests for allometry. Remarkably, there are two possible scenarios for isometry in this case. The first is isotropic variation of the landmark coordinate data, which has been extensively used as a null model in geometric morphometrics, including for the justification of how the Procrustes form space is set up (Mitteroecker et al. 2004), but it is an unrealistic model for biological data. Under this model, which implies equal amounts of variation in all dimensions of the form space, the directions of PCs are determined only by sampling error and are therefore meaningless. In a different null model, the variation for size exceeds the variation for the shape variables, as it is usually found for biological data, but size is independent of shape. Under this model, the PC1 is expected to have a coefficient of 1.0 for log-transformed centroid size (or equivalently −1.0, as the sign is arbitrary) and zero coefficients for all the shape variables. In other words, under this model, the PC1 is aligned perfectly with the axis of log-centroid size. In principle, this can be tested with the same method that Jolicoeur (1963) proposed for testing allometry against the null hypothesis of isometry for traditional morphometric data—the only difference is the PC1 coefficients expected under that null hypothesis. Implementations of this test using permutation approaches are also feasible. Intriguingly, it seems that this approach for testing allometry in Procrustes form space has not been used so far. Instead of formal statistical tests, existing studies using Procrustes form space have tended to present visualizations of the changes along the PC1 axis as graphical evidence for allometry (Mitteroecker et al. 2004, 2013; Bulygina et al. 2006; Čápová et al. 2008; Milne et al. 2012; Cardini and Polly 2013; Bastir et al. 2015; Drake et al. 2015; Freidline et al. 2015).

In many morphometric datasets, the PC1 in Procrustes form space takes up a large proportion of the total variation, sometimes 80 % and more. This is distinctly more than the share of the total variance for which PC1 tends to account in PCAs in shape space, as is especially evident from analyses containing PCAs in both types of spaces for the same datasets (Chatzigianni and Halazonetis 2009; Freidline et al. 2012; Mitteroecker et al. 2013). The greater amounts of variation for which the PC1 accounts in analyses in the Procrustes form space reflect the contribution of log-centroid size, which is often dominant. Some studies have included plots of the PC1 scores in the Procrustes form space versus log-centroid size and have found a very good fit of the data to a straight line (Singleton et al. 2011; Milne et al. 2012). It is tempting to interpret these relations as evidence for allometry, but some caution is needed. Under the null model of isometry where a dominant component of variation of centroid size is independent of shape, the PC1 of form space will be perfectly aligned with the axis of log-centroid size and shape variation will not contribute to this PC1 at all. As a consequence, the correlation between the PC1 scores and log-centroid size will be perfect. Therefore, a strong relation between the PC1 in form space and log-centroid size indicates that size variation is a dominant factor in the data but is not evidence for allometry.

For analyses where the specimens belong to multiple groups such as different species, populations, or sexes, the analyses need to take this structure of the data into account. Several studies have compared allometric trajectories of different groups in Procrustes form space by visualizing 2D or 3D scatters of PCA scores to explore the trajectories are arranged relative to each other (Mitteroecker et al. 2004, 2005; Freidline et al. 2012; Cardini and Polly 2013; Drake et al. 2015). In principle, such comparisons can use the same tools as they have long been used for comparing allometric trajectories in traditional morphometrics (Klingenberg 1996b), including angles between PC1 vectors (Pimentel 1979; Cheverud 1982; Klingenberg and Zimmermann 1992a) or ordinations of the PC1 vectors (Klingenberg and Froese 1991; Klingenberg and Spence 1993; Gerber et al. 2008; Wilson and Sánchez-Villagra 2010). Also, for obtaining simultaneous estimates of within-group allometry in multiple groups, there are methods that have long been used in traditional morphometrics, such as multigroup PCA (Pimentel 1979; Thorpe 1983) or common principal components (Airoldi and Flury 1988; Flury 1988; Klingenberg 1996b), which can be used for data in the Procrustes form space with minor modifications. Both these techniques provide a joint estimate of allometry within all the groups and are thus similar in purpose to pooled within-group regression (Fig. 7), but they are based firmly in the Huxley–Jolicoeur framework of allometry.

For size correction of data in Procrustes form space, it is possible to project the data points onto the subspace perpendicular to the PC1. This is the classical approach of the Huxley–Jolicoeur framework (Fig. 3) and is applicable in this case. The projection (Fig. 10) separates a component of size variation and size-related shape variation, in the direction of the PC1, from variation that is unrelated to it. In the absence of allometry, if variation is isometric so that the PC1 corresponds entirely to the dominant variation in log-centroid size independent of the variation of the shape variables, the result of such a size correction is simply the separation of log-centroid size and the shape tangent space. If there is allometry, however, the PC1 axis is somewhat oblique relative to the axis of log-centroid size (Fig. 10). As a consequence, the subspace perpendicular to the PC1 also is slightly inclined relative to the shape tangent space. This means that some points in this space are higher and others are lower along the axis of log-centroid size; in other words, there is some variation of log-centroid size remaining even after size correction! This paradoxical situation results from the nature of the Procrustes form space: it clearly belongs within the Huxley–Jolicoeur framework of allometry because it contains size as an intrinsic component, and consequently, the PC1 as a best-fitting line is the only logical choice for characterizing allometry, but from its construction, form space also inherits the distinction between size and shape that is the hallmark of the Gould–Mosimann school. The paradox arises because the concepts from the two frameworks clash—most notably size in this instance.

## Allometry in conformation space

A different strategy for applying the Huxley–Mosimann approach in the context of geometric morphometrics is to use a Procrustes superimposition without the scaling step, minimizing squared differences of landmark positions only over transpositions and rotations. Instead of first separating and then combining shape and size again, as in the approach of Procrustes form space, this method never separates shape and size in the first place. This approach and the associated space have been known for a long time under a bewildering variety of different names (see also Table 1) including “size-and-shape” (Kendall 1989; Le 1995; Dryden and Mardia 1998) or also “form” (Goodall 1991; Goodall and Mardia 1991), “figure” (Ziezold 1977), or “allometry space” (Langlade et al. 2005). The name “size-and-shape” is the most established, but it is also confusing because it would be a better description for the Procrustes form space, for which size and shape are first separated and then combined again, whereas in this approach, size and shape are never distinguished or separated from each other. Other terms such as “form” and “figure” have different meanings (sometimes more than one) that are well established in statistical shape analysis or in geometric morphometrics (Goodall 1991; Rohlf 1996; Mitteroecker et al. 2013). Therefore, reluctantly, I appropriate a new word for this purpose: “conformation.” The conformation of an object encompasses all its geometric features except its position and orientation. The word “conformation” is therefore a synonym to “form,” but it is introduced specifically to distinguish it from the use of “form” in the approach based on the Procrustes form space. Every conformation (or form) has its shape and size, but these are usually not quantified on their own in the course of an analysis of conformations. The new use of the term “conformation” is fully consistent with its established use in structural biology: the absolute scale is an inherent component of the conformation of a molecule, and no steps are taken to separate size from shape in the analysis of conformations of proteins and other macromolecules.

The distance between two conformations is the square root of the sum of squared coordinate differences after a superimposition in which this sum of squared coordinate differences is minimized (Ziezold 1977; Le 1995; Dryden and Mardia 1998). The translation for the optimal superimposition brings the centroids of the landmark configurations to the same point, and the optimal rotation can be computed in the same way as the rotation for the ordinary Procrustes superimposition (Dryden and Mardia 1998). Note that the sizes of the landmark configurations matter for the computation of the distance, because the same shape difference will produce a larger squared distance between two larger conformations (for a formula based on centroid sizes and the Procrustes distance, see Dryden and Mardia 1998, p. 177). For the superimposition of multiple conformations, an iterative procedure analogous to generalized Procrustes superimposition, but without a scaling step, can be used (Ziezold 1994; Le 1995). Therefore, this superimposition can be described as a Procrustes superimposition without scaling (Goswami 2006a, b; Bensmihen et al. 2008; Feng et al. 2009; Milne and O’Higgins 2012; O’Higgins and Milne 2013; Mydlová et al. 2015).

**Fig. 11:**
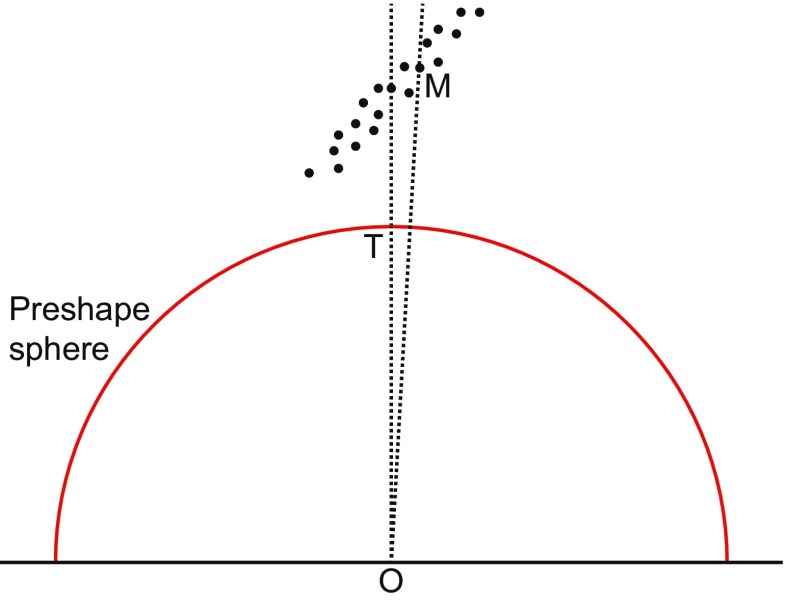
Conformation space for triangles and its relation to shape space and the sphere of aligned preshapes. The diagram shows a section through the multidimensional space of configurations aligned using translation and rotation, but with no scaling. Landmark configurations with centroid size 1.0 will be on the preshape sphere, those with greater centroid size will be outside of it (e.g., the configurations indicated by *black dots*). The *dotted lines* indicate the lines of constant shape. Note that the average shape (T) and the shape of the average conformation (M) may differ

The average conformation resulting from the superimposition procedure generally is not just a scaled version of the average shape for the same set of landmark configurations. If there is allometry, an association of size and shape, the shape of the mean conformation may differ from the mean shape (points M and T in Fig. 11). The reason for this is that larger configurations carry a greater weight in the calculations of the mean conformation because rotation of a larger configuration is more costly in terms of squared deviations between landmark positions than is the rotation of a smaller configuration. (Note: each landmark of each configuration carries the same weight per se, but the stronger weighting of bigger configurations results because the landmark coordinates of bigger configurations, after centering, tend to have greater values than those of smaller configurations.) The observation that the shape of the average conformation may differ from the average shape might be seen as a weakness of the method, but such criticism would ignore the fact that the average shape does not figure at all in the morphometric approach using conformation space (the point is *not* to separate size and shape).

**Fig. 12:**
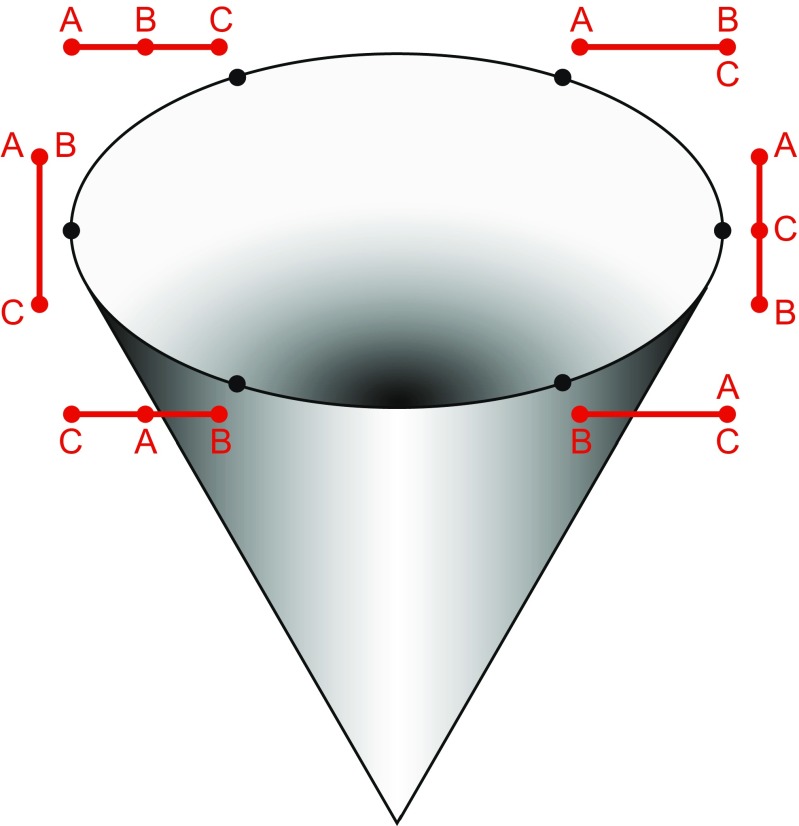
A partial view of the conformation space for collinear triangles. The edge at the top of the cone corresponds to the shape space of collinear triangles (the “equator” of Kendall’s shape space when viewed as in Fig. 5a). The cone extends down to the point that corresponds to a triangle of size zero (the totally degenerate triangle where all three corners are in the same point). The conformation space also extends further upwards as the continuation of the part of the cone shown in the diagram, for collinear triangles with greater centroid sizes

Conformation space is fairly difficult to visualize. It is useful to start with the example of triangles (Fig. 11), because it is particularly familiar in terms of its shape space. When superimposition is based on translation and rotation, the points representing the landmark configurations in conformation space are at variable distances from the origin of the coordinate system (point O in Fig. 11) and those distances are equal to the centroid sizes of the respective landmark configurations. The hemisphere of Procrustes-aligned preshapes is a special subspace in this space, as preshapes are defined as configurations that have been scaled to centroid size 1.0 (and from which variation in position has also been removed). Conformations with centroid sizes greater than 1.0 are outside this sphere, smaller configurations are inside the sphere. Because there is no scaling, the coordinates in conformation space are in the same units as the landmark coordinates, such as millimeters, centimeters, or inches. So what are the effects of changing the (arbitrary) choice of those units? The effect will be a change in scaling of the axes by a factor according to the change of units (10 for a change between centimeters and millimeters, 25.4 between inches and millimeters, etc.). In addition, such a change of units will also affect how the preshape sphere appears in relation to any data points in graphs such as Fig. 11. Because preshapes do not play any role in the analyses of conformations, however, this has no effect on the results of any analyses. Conformations with the same shape but different centroid sizes are on straight lines radiating from the origin (dotted lines originating from point O in Fig. 11). The origin also corresponds to the location of the totally degenerate configuration whose landmarks are all in the same point and which therefore has centroid size zero. Because the lines for different shapes converge in this single point, the overall structure of the conformation space is that of a cone, with sections corresponding to scaled copies of Kendall’s shape space for the appropriate number of landmarks and dimensions (Kendall 1989; Dryden and Mardia 1998). This global structure cannot be visualized directly for all triangles, but it can be shown for the subspace of collinear triangles (Fig. 12). In the traditional orientation of Kendall’s shape space for triangles that uses the two equilateral triangles as the poles, as in Fig. 5a, this subspace is the equator. Each section of the cone in Fig. 12 is a circle that contains all collinear triangles of a certain centroid size, corresponding to the distance from the apex of the cone. For each point on the section, the straight line from the vertex (and also extending beyond the current section to larger centroid sizes) contains all the conformations that have the same shape. In practice, as for the shape and form spaces, the global structure of the conformation space is less important because the focus is on a relatively small part of the whole space that is occupied by the landmark configurations in a given dataset.

**Fig. 13:**
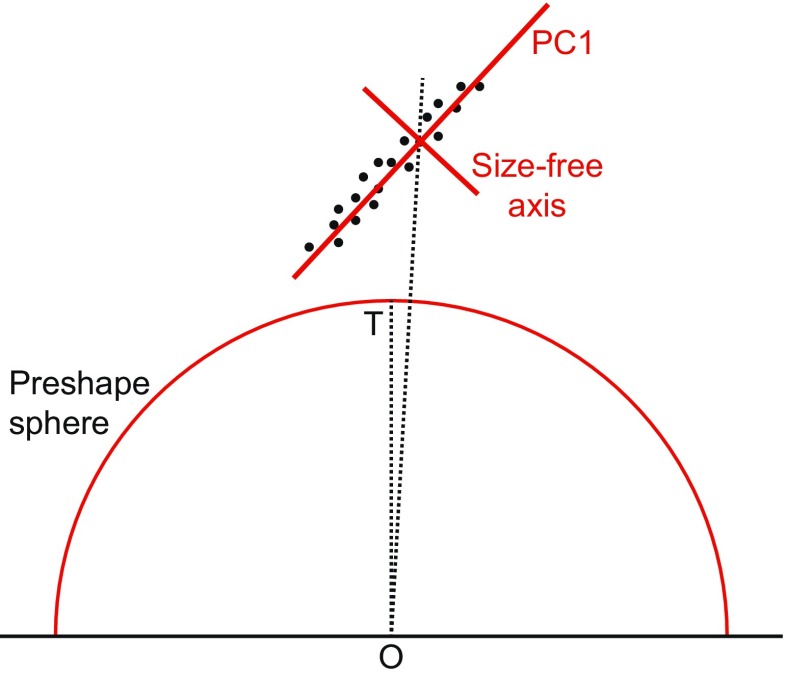
Allometry and size correction in conformation space. The data points display a trend in shape (orientation from the apex of the conformation space, point O) with increasing size (distance from the apex). The PC1 is a line of best fit to the data points in conformation space and reflects this trend. Size correction can be carried out by projecting the data points onto the subspace perpendicular to the PC1

For characterizing allometry in conformation space, it is very clear that the Huxley–Jolicoeur approach must be used. Just as for length measurements in traditional morphometrics, the landmark coordinates that characterize the conformations contain the complete morphological variation without a separation of size and shape. And just as for traditional morphometrics, for most biological datasets, size and size-related allometric variation is usually the dominant component of within-group variation in conformation space. The PC1 in the conformation space is an estimate of a linear allometric trajectory, optimal according to a least-squares criterion (Fig. 13). Empirical studies have found that the PC1s accounted for large proportions of the total variance (sometimes around 70 % or more), reflecting the substantial contribution of size and size-related variation (Langlade et al. 2005; Bensmihen et al. 2008; Rosas et al. 2012). This dominance of the PC1 is reminiscent of the results of traditional morphometric studies using length measurements.

For statistical tests of allometry, an important question is how to implement the null hypothesis of isometry. There are two straightforward choices: first, the model of isotropic variation of the landmark positions and, second, the model where centroid size can vary more than shape, but is uncorrelated to it. Under the isotropic model, there are equal amounts of variation in every dimension of the conformation space, and the direction of the PC1 is therefore determined by sampling error. In principle, tests of sphericity can be applied (Mardia et al. 1979; Pimentel 1979), but this null model is biologically very implausible. For the other null model of isometry, the PC1 is defined by a dominant component of size variation that is uncorrelated with shape variation. This implies that the PC1 coincides with the line from the mean conformation to the apex of the conformation space (rather than being at an angle to this line, as in Fig. 13). Therefore, the PC1 coefficients expected under this model of isometry are a scaled version of the mean conformation (because PC coefficients are scaled so that their squares sum up to unity, the PC coefficients happen to represent the shape of the mean conformation, which is the same as the mean shape under this model). These coefficients can be used as the expectation in a test that otherwise corresponds to the classical test of multivariate allometry (Jolicoeur 1963; Pimentel 1979).

Because variation in size is such a dominant contribution to variation in conformation space for many biological datasets, the issue of size correction is as important in this context as it has been in traditional morphometrics (Burnaby 1966; Klingenberg 1996b). Because size is inherent in the conformation space, projection onto the subspace orthogonal to the PC1 is the logical and appropriate method of size correction (Fig. 13). This correction removes the dimension that contains size and size-related variation. Size corrections of this kind have been used in empirical analyses (Goswami 2006b, 2007). If the data include multiple groups of specimens, the estimation of the allometric vector should take this into account. The same techniques that have been used in the context of multivariate allometry in the tradition of the Huxley–Jolicoeur school can be used, as discussed above for Procrustes form space, both for finding simultaneous estimates of allometry within multiple groups and for comparing allometric patterns among groups (i.e., methods such as multigroup PCA, CPCA, etc.).

## Comparison of approaches

How do these three approaches to allometry compare to each other? All three methods are mathematically correct and logical implementations that share core concepts such as allometry and isometry. For data where allometric relations fit perfectly (without residuals, etc.), all three methods provide precisely compatible results; with statistical noise added to allometric relations, the agreement may not be perfect, but no fundamental differences are to be expected. The greatest difference is that between the regression approach, which is firmly in the tradition of the Gould–Mosimann school of allometry, and the other two methods, which belong to the Huxley–Jolicoeur school. The difference between the two latter methods is more subtle, and in many ways, they are equivalent and expected to give similar results in practice.

The first question facing an investigator, therefore, is whether to treat size and shape as logically separate properties of a landmark configuration or whether to drop this distinction and consider the morphological variation as one. This is, in different words, the question whether to choose the Gould–Mosimann or Huxley–Jolicoeur framework for studies of allometry. In geometric morphometrics, the Gould–Mosimann framework has been much more prevalent and has long been the only approach available, whereas the vast majority of traditional morphometric studies of multivariate allometry have used the Huxley–Jolicoeur approach. Yet, the distinction is not entirely clear-cut, because many studies that were based on the Huxley–Jolicoeur framework have made more or less informal distinctions between size and shape (for discussion, see Bookstein 1989; Klingenberg 1996b). Now, as conceptually and mathematically solid implementations of both frameworks are available, the decisive factor should be whether the focus of interest is on shape per se or on the total morphological variation without a distinction of shape and size (for a similar recommendation, see also Mitteroecker et al. 2013).

There is one situation where the two frameworks may differ markedly: in the effects of size correction when there is little variation in size or size-related variation. If there is little variation in size, the multivariate regression of shape on size yields a vector of regression coefficients that are all close to zero. Using this vector to compute residuals makes very little difference to the data: because the regression coefficients are very small, the predicted component of variation is also small and therefore the residual variation is nearly the same as the total variation. Performing a size correction, not really necessary in this situation, has little or no effect on the data. By contrast, using the PC1 (within groups, if appropriate) as an estimate of allometry and projecting the data onto the subspace orthogonal to the PC1 does have an effect in this situation. With little variation of size or size-related variation, the PC1 reflects variation from other origins, but still is associated with the dominant feature of variation. If that dominant feature of variation is not related to allometry, “size correction” based on eliminating variation in the direction of the PC1 removes some different aspect of the data. Removing an entire dimension inevitably does affect the data, particularly so because the direction of the PC1 (possibly within groups) is a major component of variation. For that reason, before any size correction is carried out with the methods of the Huxley–Jolicoeur school, it is important to double-check whether the PC1 is indeed a component of size and size-related variation and whether there is a substantial amount of size variation. This assumption is met in many or perhaps even most biological datasets, but there is no guarantee for this to be true in general.

The two main frameworks for studying allometry differ in how size and shape are treated: the Gould–Mosimann school focuses on a phenotypic space containing only shape and uses size as an external factor, whereas the methods of the Huxley–Jolicoeur school use a phenotypic space including both size and shape. Therefore, the phenotypic spaces in the Huxley–Jolicoeur framework contain an additional dimension, containing information on size in some manner. If a size correction is performed, however, this extra dimension is removed and the difference between the phenotypic spaces is diminished. The remaining differences depend on how strong allometry is: for weak allometry, where size accounts only for a minor proportion of the total shape variation, the difference between the two approaches usually is fairly subtle. For the limiting case of isometry (with a dominant component of size variation independent of shape), the phenotype space after size correction reduces to the shape tangent space, precisely for the Procrustes form space and approximately for conformation space. For many biological datasets, therefore, the analyses of allometry using the different methods should provide results that are mutually consistent.

For the Huxley–Jolicoeur framework, a further question is how the Procrustes form space and conformation space differ from each other and whether one of them is to be preferred over the other. The biggest difference between these two methods, which has not been discussed before, is the global structure of the two spaces. Because Procrustes form spaces require a shape tangent space, each of them can cover only a limited range of shapes surrounding the tangent point, usually the mean shape of an empirical dataset. By contrast, the conformation space accommodates all possible conformations with a given number of landmarks and dimensionality. In practice, however, biological datasets cover only limited ranges of forms, so that it is unlikely that the difference in global structure of spaces makes a substantial difference to empirical studies. For the null model of a small amount of isotropic variation of landmark positions (i.e., with the amount of variation around the mean of each landmark much smaller than the distances among landmarks), which has been widely used in morphometrics, the two spaces are equivalent up to scaling by the mean centroid size. For the Procrustes form space, variation is still isotropic and the shape variables are scaled to unit centroid size because they are derived from the shape tangent space, whereas the use of log-centroid size is equivalent to dividing by mean size (Mitteroecker et al. 2004, Appendix). Likewise, the model of small isotropic variation also yields isotropic variation in conformation space (Le 1994), but at the original scale of the landmark coordinates. Under that null model, both methods therefore behave in an appropriate and equivalent manner.

Overall, therefore, all three methods are broadly compatible and should provide results that are comparable in empirical studies. The tree approaches differ in the style of how they characterize morphological variation and allometry. This should be useful for researchers who prefer one way of thinking or the other. Nevertheless, because the methods generally are compatible, the results from analyses should be interpretable across the spectrum of approaches, encouraging exchange of empirical findings among allometric analyses. Allometry has long been an important focus for studies of evolution and development, and the methods now available in geometric morphometrics are flexible and powerful tools for investigating the evolution of morphological structures and its developmental and genetic basis (Klingenberg 2010).

## References

[CR1] Adams DC, Nistri A (2010). Ontogenetic convergence and evolution of foot morphology in European cave salamanders (Family: Plethodontidae). BMC Evol Biol.

[CR2] Adams DC, Rohlf FJ, Slice DE (2013). A field comes of age: geometric morphometrics in the 21st century. Hystrix.

[CR3] Airoldi J-P, Flury B (1988). An application of common principal component analysis to cranial morphometry of *Microtus californicus* and *M. orchogaster* (Mammalia, Rodentia). J Zool (Lond).

[CR4] Barbeito-Andrés J, Ventrice F, Anzelmo M, Pucciarelli HM, Sardi ML (2015). Developmental covariation of human vault and base throughout postnatal ontogeny. Ann Anat.

[CR5] Bastir M, O’Higgins P, Rosas A (2007). Facial ontogeny in Neanderthals and modern humans. Proc R Soc Lond B Biol Sci.

[CR6] Bastir M (2015). The relevance of the first ribs of the El Sidrón site (Asturias, Spain) for the understanding of the Neandertal thorax. J Hum Evol.

[CR7] Baur H, Leuenberger C (2011). Analysis of ratios in multivariate morphometry. Syst Biol.

[CR8] Bensmihen S, Hanna AI, Langlade NB, Micol JL, Bangham A, Coen E (2008). Mutational spaces for leaf shape and size. HFSP J.

[CR9] Berge C, Kazmierczak J-B (1986). Effects of size and locomotor adaptations on the hominid pelvis: evaluation of australopithecine bipedality with a new multivariate method. Folia Primatol.

[CR10] Birch JM (1999). Skull allometry in the marine toad, *Bufo marinus*. J Morphol.

[CR11] Boitard M, Lefebvre J, Solignac M (1982). Analyse en composantes principales de la variabilité de taille, de croissance et de conformation des espèces du complexe *Jaera albifrons* (Crustacés Isopodes). Cah Biol Mar.

[CR12] Bolzan DP, Pessôa LM, Peracchi AL, Strauss RE (2015). Allometric patterns and evolution in Neotropical nectar-feeding bats (Chiroptera, Phyllostomidae). Acta Chiropt.

[CR13] Bookstein FL (1986). Size and shape spaces for landmark data in two dimensions (with comments and rejoinder). Stat Sci.

[CR14] Bookstein FL (1989). “Size and shape”: a comment on semantics. Syst Zool.

[CR15] Bookstein FL (1991). Morphometric tools for landmark data: geometry and biology.

[CR16] Bookstein FL, Chernoff B, Elder RL, Humphries JM, Jr., Smith GR, Strauss RE (1985) Morphometrics in evolutionary biology: the geometry of size and shape change, with examples from fishes, vol Special publication 15. Academy of Natural Sciences of Philadelphia, Philadelphia

[CR17] Breuker CJ, Patterson JS, Klingenberg CP (2006). A single basis for developmental buffering of *Drosophila* wing shape. PLoS ONE.

[CR18] Bulygina E, Mitteroecker P, Aiello L (2006). Ontogeny of facial dimorphism and patterns of individual development within one human population. Am J Phys Anthropol.

[CR19] Burnaby TP (1966). Growth-invariant discriminant functions and generalized distances. Biometrics.

[CR20] Cadima JFCL, Jolliffe IT (1996). Size- and shape-related principal component analysis. Biometrics.

[CR21] Calder WA (1984). Size, function, and life history.

[CR22] Čápová M, Zlatnická I, Kováč V, Katina S (2008). Ontogenetic variability in the external morphology of monkey goby, *Neogobius fluviatilis* (Pallas, 1814) and its relevance to invasion potential. Hydrobiologia.

[CR23] Cardini A, Polly PD (2013). Larger mammals have longer faces because of size-related constraints on skull form. Nat Commun.

[CR24] Chatzigianni A, Halazonetis DJ (2009). Geometric morphometric evaluation of cervical vertebrae shape and its relationship to skeletal maturation. Am J Orthod Dentofacial Orthop.

[CR25] Cheverud JM (1982). Relationships among ontogenetic, static, and evolutionary allometry. Am J Phys Anthropol.

[CR26] Cobb SN, O’Higgins P (2007). The ontogeny of sexual dimorphism in the facial skeleton of the African apes. J Hum Evol.

[CR27] Cock AG (1966). Genetical aspects of metrical growth and form in animals. Q Rev Biol.

[CR28] Creighton GK, Strauss RE (1986). Comparative patterns of growth and development in cricetine rodents and the evolution of ontogeny. Evolution.

[CR29] Darroch JN, Mosimann JE (1985). Canonical and principal components of shape. Biometrika.

[CR30] Davies RG, Brown V (1972). A multivariate analysis of postembryonic growth in two species of *Ectobius* (Dictyoptera: Blattidae). J Zool.

[CR31] Drake AG, Klingenberg CP (2008). The pace of morphological change: historical transformation of skull shape in St. Bernard dogs. Proc R Soc Lond B Biol Sci.

[CR32] Drake AG, Coquerelle M, Colombeau G (2015). 3D morphometric analysis of fossil canid skulls contradicts the suggested domestication of dogs during the late Paleolithic. Sci Rep.

[CR33] Dryden IL, Mardia KV (1992). Size and shape analysis of landmark data. Biometrika.

[CR34] Dryden IL, Mardia KV (1998). Statistical shape analysis.

[CR35] Fadda C, Leirs H (2009). The role of growth stop as a morphogenetic factor in *Mastomys natalensis* (Rodentia: Muridae). Biol J Linn Soc.

[CR36] Feng X (2009). Evolution of allometry in *Antirrhinum*. Plant Cell.

[CR37] Flury B (1988). Common principal components and related multivariate models.

[CR38] Frédérich B, Vandewalle P (2011). Bipartite life cycle of coral reef fishes promotes increasing shape disparity of the head skeleton during ontogeny: an example from damselfishes (Pomacentridae). BMC Evol Biol.

[CR39] Frédérich B, Adriaens D, Vandewalle P (2008). Ontogenetic shape changes in Pomacentridae (Teleostei, Perciformes) and their relationships with feeding strategies: a geometric morphometric approach. Biol J Linn Soc.

[CR40] Freidline SE, Gunz P, Harvati K, Hublin J-J (2012). Middle Pleistocene human facial morphology in an evolutionary and developmental context. J Hum Evol.

[CR41] Freidline SE, Gunz P, Hublin J-J (2015). Ontogenetic and static allometry in the human face: contrasting Khoisan and Inuit. Am J Phys Anthropol.

[CR42] Frost SR, Marcus LF, Bookstein FL, Reddy DP, Delson E (2003). Cranial allometry, phylogeography, and systematics of large-bodied papionins (Primates: Cercopithecinae) inferred from geometric morphometric analysis of landmark data. Anat Rec.

[CR43] Gerber S, Eble GJ, Neige P (2008). Allometric space and allometric disparity: a developmental perspective in the macroevolutionary analysis of morphological disparity. Evolution.

[CR44] Gibson AR, Baker AJ, Moeed A (1984). Morphometric variation in introduced populations of the common myna (*Acridotheres tristis*): an application of the jackknife to principal component analysis. Syst Zool.

[CR45] Gidaszewski NA, Baylac M, Klingenberg CP (2009). Evolution of sexual dimorphism of wing shape in the *Drosophila melanogaster* subgroup. BMC Evol Biol.

[CR46] Golubović A, Tomović L, Ivanović A (2015). Geometry of self righting—case of Hermann’s tortoises. Zool Anz.

[CR47] Gonzalez PN, Perez SI, Bernal V (2010). Ontogeny of robusticity of craniofacial traits in modern humans: a study of South American populations. Am J Phys Anthropol.

[CR48] Gonzalez PN, Perez SI, Bernal V (2011). Ontogenetic allometry and cranial shape diversification among human populations from South America. Anat Rec.

[CR49] Good P (2000). Permutation tests: a practical guide to resampling methods for testing hypotheses.

[CR50] Goodall CR (1991). Procrustes methods in the statistical analysis of shape. J R Statist Soc B.

[CR51] Goodall CR, Mardia KV (1991). A geometrical derivation of the shape density. Adv Appl Prob.

[CR52] Goodall CR, Mardia KV (1993). Multivariate aspects of shape theory. Ann Stat.

[CR53] Goswami A (2006). Cranial modularity shifts during mammalian evolution. Am Nat.

[CR54] Goswami A (2006). Morphological integration in the carnivoran skull. Evolution.

[CR55] Goswami A (2007). Phylogeny, diet and cranial integration in australodelphian marsupials. PLoS ONE.

[CR56] Gould SJ (1966). Allometry and size in ontogeny and phylogeny. Biol Rev.

[CR57] Huxley JS (1924). Constant differential growth-ratios and their significance. Nature.

[CR58] Huxley JS (1932) Problems of relative growth. Reprinted 1993 edn. Johns Hopkins University Press, Baltimore

[CR59] Huxley JS, Teissier G (1936). Terminology of relative growth. Nature.

[CR60] Ivanović A, Kalezić ML (2010). Testing the hypothesis of morphological integration on a skull of a vertebrate with a biphasic life cycle: a case study of the alpine newt. J Exp Zool B Mol Dev Evol.

[CR61] Johnson RA, Wichern DW (1988). Applied multivariate statistical analysis.

[CR62] Jojić V, Blagojević J, Vujošević M (2012). Two-module organization of the mandible in the yellow-necked mouse: a comparison between two different morphometric approaches. J Evol Biol.

[CR63] Jolicoeur P (1963). The multivariate generalization of the allometry equation. Biometrics.

[CR64] Jolicoeur P, Mosimann JE (1960). Size and shape variation in the painted turtle: a principal component analysis. Growth.

[CR65] Jolliffe IT (2002). Principal component analysis.

[CR66] Jones CS (1992). Comparative ontogeny of a wild cucurbit and its derived cultivar. Evolution.

[CR67] Jungers WL, Falsetti AB, Wall CE (1995). Shape, relative size, and size-adjustments in morphometrics. Yearb Phys Anthropol.

[CR68] Kazmierczak JB (1985). Analyse logarithmique: deux exemples d’application. Rev Stat Appl.

[CR69] Kendall DG (1984). Shape manifolds, procrustean metrics, and complex projective spaces. Bull Lond Math Soc.

[CR70] Kendall DG (1989). A survey of the statistical theory of shape. Stat Sci.

[CR71] Kendall DG, Barden D, Carne TK, Le H (1999). Shape and shape theory.

[CR72] Klingenberg CP (1996). Individual variation of ontogenies: a longitudinal study of growth and timing. Evolution.

[CR73] Klingenberg CP, Marcus LF, Corti M, Loy A, Naylor GJP, Slice DE (1996). Multivariate allometry. Advances in morphometrics.

[CR74] Klingenberg CP (1998). Heterochrony and allometry: the analysis of evolutionary change in ontogeny. Biol Rev.

[CR75] Klingenberg CP (2009). Morphometric integration and modularity in configurations of landmarks: tools for evaluating a-priori hypotheses. Evol Dev.

[CR76] Klingenberg CP (2010). Evolution and development of shape: integrating quantitative approaches. Nat Rev Genet.

[CR77] Klingenberg CP (2013). Cranial integration and modularity: insights into evolution and development from morphometric data. Hystrix.

[CR78] Klingenberg CP (2013). Visualizations in geometric morphometrics: how to read and how to make graphs showing shape changes. Hystrix.

[CR79] Klingenberg CP (2014). Studying morphological integration and modularity at multiple levels: concepts and analysis. Philos Trans R Soc Lond B Biol Sci.

[CR80] Klingenberg CP (2015). Analyzing fluctuating asymmetry with geometric morphometrics: concepts, methods, and applications. Symmetry.

[CR81] Klingenberg CP, Ekau W (1996). A combined morphometric and phylogenetic analysis of an ecomorphological trend: pelagization in Antarctic fishes (Perciformes: Nototheniidae). Biol J Linn Soc.

[CR82] Klingenberg CP, Froese R (1991). A multivariate comparison of allometric growth patterns. Syst Zool.

[CR83] Klingenberg CP, Marugán-Lobón J (2013). Evolutionary covariation in geometric morphometric data: analyzing integration, modularity and allometry in a phylogenetic context. Syst Biol.

[CR84] Klingenberg CP, Spence JR (1993). Heterochrony and allometry: lessons from the water strider genus *Limnoporus*. Evolution.

[CR85] Klingenberg CP, Zimmermann M (1992). Static, ontogenetic, and evolutionary allometry: a multivariate comparison in nine species of water striders. Am Nat.

[CR86] Klingenberg CP, Zimmermann M (1992). Dyar’s rule and multivariate allometric growth in nine species of waterstriders (Heteroptera, Gerridae). J Zool.

[CR87] Klingenberg CP, Badyaev AV, Sowry SM, Beckwith NJ (2001). Inferring developmental modularity from morphological integration: analysis of individual variation and asymmetry in bumblebee wings. Am Nat.

[CR88] Klingenberg CP, Mebus K, Auffray J-C (2003). Developmental integration in a complex morphological structure: how distinct are the modules in the mouse mandible?. Evol Dev.

[CR89] Klingenberg CP, Duttke S, Whelan S, Kim M (2012). Developmental plasticity, morphological variation and evolvability: a multilevel analysis of morphometric integration in the shape of compound leaves. J Evol Biol.

[CR90] Kölliker-Ott UM, Blows MW, Hoffmann AA (2003). Are wing size, wing shape and asymmetry related to field fitness of *Trichoramma* egg parasitoids?. Oikos.

[CR91] Langlade NB (2005). Evolution through genetically controlled allometry space. Proc Natl Acad Sci U S A.

[CR92] Larson PM (2004). Chondrocranial morphology and ontogenetic allometry in larval *Bufo americanus* (Anura, Bufonidae). Zoomorphol (Berl).

[CR93] Lazić M, Carretero MA, Crnobrnja-Isailović J, Kaliontzopoulou A (2015). Effects of environmental disturbance on phenotypic variation: an integrated assessment of canalization, developmental stability, modularity, and allometry in lizard head shape. Am Nat.

[CR94] Le H (1994). Brownian motions on shape and size-and-shape spaces. J Appl Prob.

[CR95] Le H (1995). Mean size-and-shapes and mean shapes: a geometric point of view. Adv Appl Prob.

[CR96] Leamy L, Bradley D (1982). Static and growth allometry of morphometric traits in randombred house mice. Evolution.

[CR97] Leamy L, Thorpe RS (1984). Morphometric studies in inbred and hybrid house mice. Heterosis, homeostasis, and heritability of size and shape. Biol J Linn Soc.

[CR98] Lele S, Richtsmeier JT (1991). Euclidean distance matrix analysis: a coordinate-free approach for comparing biological shapes using landmark data. Am J Phys Anthropol.

[CR99] Lessa EP, Patton JL (1989). Structural constraints, recurrent shapes, and allometry in pocket gophers (genus *Thomomys*). Biol J Linn Soc.

[CR100] Ljubisavljević K, Urošević A, Aleksić I, Ivanović A (2010). Sexual dimorphism of skull shape in a lacertid lizard species (*Podarcis* spp., *Dalmatolacerta* sp., *Dinarolacerta* sp.) revealed by geometric morphometrics. Zoology (Jena).

[CR101] Loy A, Cataudella S, Corti M, Marcus LF, Corti M, Loy A, Naylor GJP, Slice DE (1996). Shape changes during the growth of the sea bass, *Dicentrarchus labrax* (Teleostea: Perciformes), in relation to different rearing conditions. Advances in morphometrics.

[CR102] Loy A, Mariani L, Bertelletti M, Tunesi L (1998). Visualizing allometry: geometric morphometrics in the study of shape changes in the early stages of the two-banded sea bream, *Diplodus vulgaris* (Perciformes, Sparidae). J Morphol.

[CR103] Ludoški J, Djurakic M, Pastor B, Martínez-Sánchez AI, Rojo S, Milankov V (2014). Phenotypic variation of the housefly, *Musca domestica*: amounts and patterns of wing shape asymmetry in wild populations and laboratory colonies. Bull Entomol Res.

[CR104] Malhotra A, Thorpe RS (1997). Size and shape variation in a Lesser Antillean anole, *Anolis oculatus* (Sauria: Iguanidae) in relation to habitat. Biol J Linn Soc.

[CR105] Marcus LF, Bello E, García-Valdecasas A (1993). Contributions to morphometrics.

[CR106] Marcus LF, Corti M, Loy A, Naylor GJP, Slice DE (1996). Advances in morphometrics.

[CR107] Marcus LF, Hingst-Zaher E, Zaher H (2000). Application of landmark morphometrics to skulls representing the orders of living mammals. Hystrix.

[CR108] Mardia KV, Kent JT, Bibby JM (1979). Multivariate analysis.

[CR109] Mardia KV, Coombes A, Kirkbride J, Linney A, Bowie JL (1996). On statistical problems with face identification from photographs. J Appl Stat.

[CR110] Martínez-Abadías N, Heuzé Y, Wang Y, Jabs EW, Aldridge K, Richtsmeier JT (2011). FGF/FGFR signaling coordinates skull development by modulating magnitude of morphological integration: evidence from Apert syndrome mouse models. PLoS ONE.

[CR111] Martínez-Vargas J, Muñoz-Muñoz F, Medarde N, López-Fuster MJ, Ventura J (2014). Effect of chromosomal reorganizations on morphological covariation of the mouse mandible: insights from a Robertsonian system of *Mus musculus domesticus*. Front Zool.

[CR112] Martín-Serra A, Figueirido B, Palmqvist P (2014). A three-dimensional analysis of morphological evolution and locomotor performance of the carnivoran forelimb. PLoS ONE.

[CR113] McCoy MW, Bolker BM, Osenberg CW, Miner BG, Vonesh JR (2006). Size correction: comparing morphological traits among populations and environments. Oecolog (Berl).

[CR114] Milne N, O’Higgins P (2012). Scaling of form and function in the xenarthran femur: a 100-fold increase in body mass is mitigated by repositioning of the third trochanter. Proc R Soc Lond B Biol Sci.

[CR115] Milne N, Toledo N, Vizcaíno SF (2012). Allometric and group differences in the xenarthran femur. J Mamm Evol.

[CR116] Mitteroecker P, Bookstein FL (2007). The conceptual and statistical relationship between modularity and morphological integration. Syst Biol.

[CR117] Mitteroecker P, Gunz P (2009). Advances in geometric morphometrics. Evol Biol.

[CR118] Mitteroecker P, Gunz P, Bernhard M, Schaefer K, Bookstein FL (2004). Comparison of cranial ontogenetic trajectories among great apes and humans. J Hum Evol.

[CR119] Mitteroecker P, Gunz P, Bookstein FL (2005). Heterochrony and geometric morphometrics: a comparison of cranial growth in *Pan paniscus* versus *Pan troglodytes*. Evol Dev.

[CR120] Mitteroecker P, Gunz P, Windhager S, Schaefer K (2013). A brief review of shape, form, and allometry in geometric morphometrics, with applications to human facial morphology. Hystrix.

[CR121] Monteiro LR (1999). Multivariate regression models and geometric morphometrics: the search for causal factors in the analysis of shape. Syst Biol.

[CR122] Monteiro LR, dos Reis SF (1999). Princípios de morfometria geométrica.

[CR123] Morimoto N, Ogihara N, Katayama K, Shiota K (2008). Three-dimensional ontogenetic shape changes in the human cranium during the fetal period. J Anat.

[CR124] Mosimann JE (1970). Size allometry: size and shape variables with characterizations of the lognormal and generalized gamma distributions. J Am Stat Assoc.

[CR125] Mosimann JE, James FC (1979). New statistical methods for allometry with application to Florida red-winged blackbirds. Evolution.

[CR126] Murta-Fonseca RA, Fernandes DS (2016). The skull of *Hydrodynastes gigas* (Duméril, Bibron & Duméril, 1854) (Serpentes: Dipsadidae) as a model of snake ontogenetic allometry inferred by geometric morphometrics. Zoomorphol (Berl).

[CR127] Mydlová M, Dupej J, Koudelová J, Velemínská J (2015) Sexual dimorphism of facial appearance in ageing human adults: a cross-sectional study. Forensic Sci Int 257:519.e1–519.e9 10.1016/j.forsciint.2015.09.008 26548377

[CR128] O’Higgins P, Jones N (1998). Facial growth in *Cercocebus torquatus*: an application of three-dimensional geometric morphometric techniques to the study of morphological variation. J Anat.

[CR129] O’Higgins P, Milne N (2013). Applying geometric morphometrics to compare changes in size and shape arising from finite elements analyses. Hystrix.

[CR130] Openshaw GH, Keogh JS (2014). Head shape evolution in monitor lizards (*Varanus*): interactions between extreme size disparity, phylogeny and ecology. J Evol Biol.

[CR131] Oxnard CE (1974). Functional inferences from morphometrics: problems posed by uniqueness and diversity among the primates. Syst Zool.

[CR132] Patterson JS, Schofield CJ, Dujardin J-P, Miles MA (2001). Population morphometric analysis of the tropicopolitan bug *Triatoma rubrofasciata* and relationships with Old World species of *Triatoma*: evidence of New World ancestry. Med Vet Entomol.

[CR133] Pearson K (1901). On lines and planes of closest fit to systems of points in space. Philos Mag J Sci.

[CR134] Pélabon C, Bolstad GH, Egset CK, Cheverud JM, Pavlicev M, Rosenqvist G (2013). On the relationship between ontigenetic and static allometry. Am Nat.

[CR135] Pimentel RA (1979). Morphometrics: the multivariate analysis of biological data.

[CR136] Pitman EJG (1937). Significance tests which may be applied to samples from any populations. II. The correlation coefficient test. J R Statist Soc B.

[CR137] Ponssa ML, Candioti MFV (2012). Patterns of skull development in anurans: size and shape relationship during postmetamorphic cranial ontogeny in five species of the *Leptodactylus fuscus* group (Anura: Leptodactylidae). Zoomorphol (Berl).

[CR138] Reyment RA, Blackith RE, Campbell NA (1984). Multivariate morphometrics.

[CR139] Richtsmeier JT, Lele S (1993). A coordinate-free approach to the analysis of growth patterns: models and theoretical considerations. Biol Rev.

[CR140] Rodríguez-Mendoza R, Muñoz M, Saborido-Rey F (2011). Ontogenetic allometry of the bluemouth, *Helicolenus dactylopterus dactylopterus* (Teleostei: Scorpaenidae), in the Northeast Atlantic and Mediterranean based on geometric morphometrics. Hydrobiologia.

[CR141] Rohlf FJ (1990). Morphometrics. Annu Rev Ecol Syst.

[CR142] Rohlf FJ, Marcus LF, Corti M, Loy A, Naylor GJP, Slice DE (1996). Morphometric spaces, shape components and the effects of linear transformations. Advances in morphometrics.

[CR143] Rohlf FJ (1999). Shape statistics: procrustes superimpositions and tangent spaces. J Classif.

[CR144] Rohlf FJ (2000). On the use of shape spaces to compare morphometric methods. Hystrix.

[CR145] Rohlf FJ, Bookstein FL (1987). A comment on shearing as a method for “size correction”. Syst Zool.

[CR146] Rohlf FJ, Bookstein FL (eds) (1990) Proceedings of the Michigan morphometrics workshop. Special publication no 2. University of Michigan Museum of Zoology, Ann Arbor, MI

[CR147] Rohlf FJ, Marcus LF (1993). A revolution in morphometrics. Trends Ecol Evol.

[CR148] Rosas A, Bastir M (2002). Thin-plate spline analysis of allometry and sexual dimorphism in the human craniofacial complex. Am J Phys Anthropol.

[CR149] Rosas U, Zhou RW, Castillo G, Collazo-Ortega M (2012). Developmental reaction norms for water stressed seedlings of succulent cacti. PLoS ONE.

[CR150] Sallan LC, Friedman M (2012). Heads or tails: staged diversification in vertebrate evolutionary radiations. Proc Roy Soc Lond Biol Sci.

[CR151] Sardi ML, Ramírez Rozzi FV (2012). Different cranial ontogeny in Europeans and Southern Africans. PLoS ONE.

[CR152] Sardi ML, Ventrice F, Ramírez Rozzi F (2007). Allometries throughout the late prenatal and early postnatal human craniofacial ontogeny. Anat Rec.

[CR153] Schmidt-Nielsen K (1984). Scaling: why is animal size so important?.

[CR154] Shea BT (1985). Bivariate and multivariate growth allometry: statistical and biological considerations. J Zool.

[CR155] Sherratt E, Gower DJ, Klingenberg CP, Wilkinson M (2014). Evolution of cranial shape in caecilians (Amphibia: Gymnophiona). Evol Biol.

[CR156] Sidlauskas BL, Mol JH, Vari RP (2011). Dealing with allometry in linear and geometric morphometrics: a taxonomic case study in the *Leporinus cylindriformis* group (Characiformes: Anostomidae) with description of a new species from Suriname. Zool J Linn Soc.

[CR157] Singleton M (2002). Patterns of cranial shape variation in the Papionini (Primates: Cercopithecinae). J Hum Evol.

[CR158] Singleton M, Rosenberger AL, Robinson C, O’Neill R (2011). Allometric and metameric shape variation in *Pan* mandibular molars: a digital morphometric analysis. Anat Rec.

[CR159] Small CG (1996). The statistical theory of shape.

[CR160] Smith MF, Patton JL (1988). Subspecies of pocket gophers: causal bases for geographic differentiation in *Thomomys bottae*. Syst Zool.

[CR161] Sneath PHA, Sokal RR (1973). Numerical taxonomy: the principles and practice of numerical classification.

[CR162] Solignac M, Cariou M-L, Wimitzky M (1990). Variability, specificity and evolution of growth gradients in the species complex *Jaera albifrons* (Isopoda, Asellota). Crustacean (Leiden).

[CR163] Somers KM (1986). Multivariate allometry and removal of size with principal components analysis. Syst Zool.

[CR164] Strelin MM, Benitez-Vieyra SM, Fornoni J, Klingenberg CP, Cocucci AA (2016) Exploring the ontogenetic scaling hypothesis during the diversification of pollination syndromes in *Caiophora* (Loasaceae, subfam. Loasoideae). Ann Bot doi:10.1093/aob/mcw1035 10.1093/aob/mcw035 PMC4845809 27056974

[CR165] Thorpe RS, Felsenstein J (1983). A review of the numerical methods for recognising and analysing racial differentiation. Numerical taxonomy.

[CR166] Timm NH (2002). Applied multivariate analysis.

[CR167] Urošević A, Ljubisavljević K, Ivanović A (2013). Patterns of cranial ontogeny in lacertid lizards: morphological and allometric disparity. J Evol Biol.

[CR168] Viscosi V (2015). Geometric morphometrics and leaf phenotypic plasticity: assessing fluctuating asymmetry and allometry in European white oaks (*Quercus*). Bot J Linn Soc.

[CR169] Viscosi V, Cardini A (2011). Leaf morphology, taxonomy and geometric morphometrics: a simplified protocol for beginners. PLoS ONE.

[CR170] Walker JA, Marcus LF, Bello E, García-Valdecasas A (1993). Ontogenetic allometry of threespine stickleback body form using landmark-based morphometrics. Contributions to morphometrics.

[CR171] Watanabe A, Slice DE (2014). The utility of cranial ontogeny for phylogenetic inference: a case study in crocodylians using geometric morphometrics. J Evol Biol.

[CR172] Weber GW, Bookstein FL (2011). Virtual anthropology: a guide to a new interdisciplinary field.

[CR173] Weisbecker V (2012). Distortion in formalin-fixed brains: using geometric morphometrics to quantify the worst-case scenario in mice. Brain Struct Funct.

[CR174] Weisensee KE, Jantz RL (2011). Secular change in craniofacial morphology of the Portuguese using geometric morphometrics. Am J Phys Anthropol.

[CR175] White J (2009). Geometric morphometric investigation of molar shape diversity in modern lemurs and lorises. Anat Rec.

[CR176] Wilson LAB (2013). Allometric disparity in rodent evolution. Ecol Evol.

[CR177] Wilson LAB, Sánchez-Villagra MR (2010). Diversity trends and their ontogenetic basis: an exploration of allometric disparity in rodents. Proc R Soc Lond B Biol Sci.

[CR178] Zelditch ML, Sheets HD, Fink WL (2000). Spatiotemporal reorganization of growth rates in the evolution of ontogeny. Evolution.

[CR179] Zelditch ML, Sheets HD, Fink WL (2003). The ontogenetic dynamics of shape disparity. Paleobiol.

[CR180] Zelditch ML, Swiderski DL, Sheets HD (2012). Geometric morphometrics for biologists: a primer.

[CR181] Ziezold H (1977) On expected figures and a strong law of large numbers for random elements in quasi-metric spaces. In: Transactions of the seventh Prague conference on information theory, statistical decision functions and random processes, vol A. Reidel, Dordrecht, Holland, pp 591–602

[CR182] Ziezold H (1994). Mean figures and mean shapes applied to biological figure and shape distributions in the plane. Biom J.

[CR183] Zollikofer CPE, Ponce de León MS (2002). Visualizing patterns of craniofacial shape variation in *Homo sapiens*. Proc R Soc Lond B Biol Sci.

